# Assembly theory explains and quantifies selection and evolution

**DOI:** 10.1038/s41586-023-06600-9

**Published:** 2023-10-04

**Authors:** Abhishek Sharma, Dániel Czégel, Michael Lachmann, Christopher P. Kempes, Sara I. Walker, Leroy Cronin

**Affiliations:** 1 School of Chemistry, University of Glasgow, Glasgow, UK; 2 BEYOND Center for Fundamental Concepts in Science, Arizona State University, Tempe, AZ USA; 3 Institute of Evolution, Centre for Ecological Research, Budapest, Hungary; 4 The Santa Fe Institute, Santa Fe, NM USA; 5 School of Earth and Space Exploration, Arizona State University, Tempe, AZ USA

**Keywords:** Biological physics, Evolutionary theory

## Abstract

Scientists have grappled with reconciling biological evolution^1,2^ with the immutable laws of the Universe defined by physics. These laws underpin life’s origin, evolution and the development of human culture and technology, yet they do not predict the emergence of these phenomena. Evolutionary theory explains why some things exist and others do not through the lens of selection. To comprehend how diverse, open-ended forms can emerge from physics without an inherent design blueprint, a new approach to understanding and quantifying selection is necessary^3–5^. We present assembly theory (AT) as a framework that does not alter the laws of physics, but redefines the concept of an ‘object’ on which these laws act. AT conceptualizes objects not as point particles, but as entities defined by their possible formation histories. This allows objects to show evidence of selection, within well-defined boundaries of individuals or selected units. We introduce a measure called assembly (*A*), capturing the degree of causation required to produce a given ensemble of objects. This approach enables us to incorporate novelty generation and selection into the physics of complex objects. It explains how these objects can be characterized through a forward dynamical process considering their assembly. By reimagining the concept of matter within assembly spaces, AT provides a powerful interface between physics and biology. It discloses a new aspect of physics emerging at the chemical scale, whereby history and causal contingency influence what exists.

## Abstract (web summary)

Assembly theory conceptualizes objects as entities defined by their possible formation histories, allowing a unified language for describing selection, evolution and the generation of novelty.

## Main

In evolutionary theory, natural selection^1^ describes why some things exist and others do not^2^. Darwin’s theory of evolution and its modern synthesis point out how selection among variants in the past generates current functionality^3^, as well as a forward-looking process^4^. Neither addresses the space in which new phenotypic variants are generated. Physics can, in theory, take us from past initial conditions to current and future states. However, because physics has no functional view of the Universe, it cannot distinguish novel functional features from random fluctuations, which means that talking about true novelty is impossible in physical reductionism. Thus, the open-ended generation of novelty^5^ does not fit cleanly in the paradigmatic frameworks of either biology^6^ or physics^7^, and so must resort ultimately to randomness^8^. There have been several efforts to explore the gap between physics and evolution^9,10^. This is because a growing state space over time requires the exploration of a large combinatorial set of possibilities^11^, such as in the theory of the adjacent possible^12^. However, the search generates an unsustainable expansion in the number of configurations possible in a finite universe in finite time, and does not include selection. In addition, this approach has limited predictive power with respect to why only some evolutionary innovations happen and not others. Other efforts have studied the evolution of rules acting on other rules^13^; however, these models are abstract so it is difficult to see how they can describe—and predict—the evolution of physical objects.

Here, we introduce AT, which addresses these challenges by describing how novelty generation and selection can operate in forward-evolving processes. The framework of AT allows us to predict features of new discoveries during selection, and to quantify how much selection was necessary to produce observed objects^14,15^ without having to prespecify individuals or units of selection. In AT, objects are not considered as point particles (as in most physics), but are defined by the histories of their formation as an intrinsic property, mapped as an assembly space. The assembly space is defined as the pathway by which a given object can be built from elementary building blocks, using only recursive operations. For the shortest path, the assembly space captures the minimal memory, in terms of the minimal number of operations necessary to construct an observed object based on objects that could have existed in its past^16^. One feature of biological assemblies of objects is multiple realizability wherein biological evolution can produce functionally equivalent classes of objects with modular use of units in many different contexts. For each unit, the minimal assembly is unique and independent of its formation, and therefore accounts for multiple realizability in how it could be constructed^17,18^.

We introduce the foundations of AT and its implementation to quantify the degree of selection and evolution found in a collection of objects. Assembly is a function of two quantities: the number of copies of the observed objects and the objects’ assembly indices (an assembly index is the number of steps on a minimal path producing the object). Assembly captures the amount of memory necessary to produce a selected configuration of historically contingent objects in a manner similar to how entropy quantifies the information (or lack thereof) necessary to specify the configuration of an ensemble of point particles, but assembly differs from entropy because of its explicit dependence on the contingency in construction paths intrinsic to complex objects. We demonstrate how AT leads to a unified language for describing selection and the generation of novelty, and thereby produce a framework to unify descriptions of selection across physics and biology.

## Assembly theory

The concept of an object in AT is simple and rigorously defined. An object is finite, is distinguishable, persists over time and is breakable such that the set of constraints to construct it from elementary building blocks is quantifiable. This definition is, in some sense, opposite to standard physics, which treats objects of interest as fundamental and unbreakable (for example, the concept of ‘atoms’ as indivisible, which now applies to elementary particles). In AT, we recognize that the smallest unit of matter is typically defined by the limits of observational measurements and may not itself be fundamental. A more universal concept is to treat objects as anything that can be broken and built. This allows us to naturally account for the emergent objects produced by evolution and selection as fundamental to the theory. The concept of copy number is of foundational importance in defining a theory that accounts for selection. The more complex a given object, the less likely an identical copy can exist without selection of some information-driven mechanism that generates that object. An object that exists in multiple copies allows the signatures describing the set of constraints that built it to be measured experimentally. For example, mass spectrometry can be used to measure assembly for molecules, because it can measure how molecules are built by making bonds^19^.

## Assembly index and copy number

**Fig. 1:**
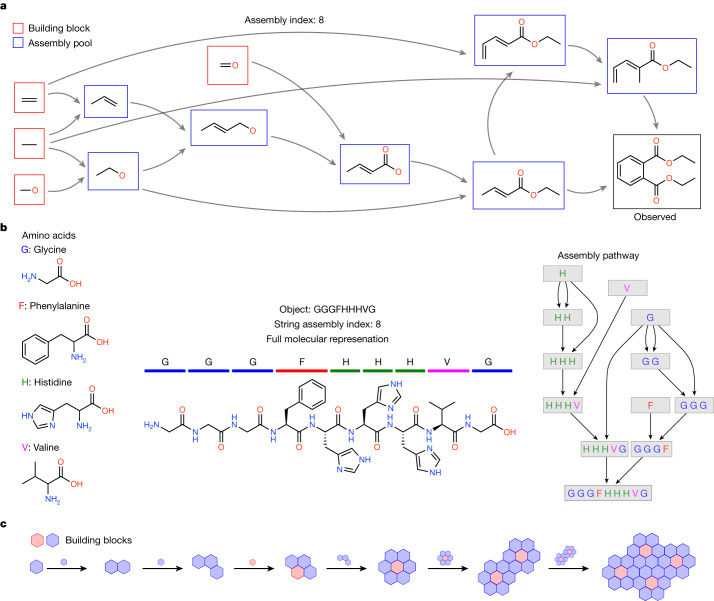
Assembly index and shortest path(s). **a**–**c**, AT is generalizable to different classes of objects, illustrated here for three different general types. **a**, Assembly pathway to construct diethyl phthalate molecule considering molecular bonds as the building blocks. The figure shows the pathway starting with the irreducible constructs to create the molecule with assembly index 8. **b**, Assembly pathway of a peptide chain by considering building blocks as strings. Left, four amino acids as building blocks. Middle, the actual object and its representation as a string. Right, assembly pathway to construct the string. **c**, Generalized assembly pathway of an object comprising discrete components.

To construct an assembly space for an object, one starts from elementary building blocks comprising that object and recursively joins these to form new structures, whereby, at each recursive step, the objects formed are added back to the assembly pool and are available for subsequent steps (Supplementary Information Sections 1 and 2). AT captures symmetry breaking arising along construction paths due to recursive use of past objects that can be combined in different ways to make new things. For any given object *i*, we can define its assembly space as all recursively assembled pathways that produce it. For each object, the most important feature is the assembly index ${a}_{i}$, which corresponds to the shortest number of steps required to generate the object from basic building blocks. This can be quantified as the length of the shortest assembly pathway that can generate the object (Fig. 1).

In chemical systems, molecular assembly theory treats bonds as the elementary operations from which molecules are constructed. The shortest path to build a given molecule can be found by breaking its bonds and then ordering its motifs in order of size, starting from atoms and moving to larger motifs by adding bonds in sequence. Given a motif generated on the path, the motif remains available for reuse. The recursivity allows identifying the shortest construction path with parts already built on that path, allowing us to quantify the minimum number of constraints, or memory size, to construct the molecule. The assembly index can be estimated from any complex discrete object with well-defined building blocks, which can be broken apart, as shown in Fig. 1. At every step, the size of the object increases by at least one. The number of total possible steps, although potentially large, is always finite for any finite object and thus the assembly index is computable in finite time. For molecules, the assembly index can be determined experimentally.

A hallmark feature of life is how complex objects are generated by evolution, of which many are functional. For example, a DNA molecule holds genetic information reliably and can be copied easily. By contrast, a random string of letters requires much information to describe it, but is not normally seen as very complex or useful. Thus far, science has not been able to find a measure that quantifies the complexity of functionality to distinguish these two cases. Here we overcome this inherent problem by pointing out another feature of the evolutionary process: the complex and functional objects it generates take many steps to make, and selection allows many identical copies of these objects. Therefore, an evolutionary process can be identified by the production of many identical, or near-identical, multistep objects. The assembly index on its own cannot detect selection, but copy number combined with the assembly index can. This approach defines a new way to measure complexity in terms of the hierarchy of causation stemming from selection at different levels.

Because we do not typically know the full assembly trajectory of an object, we instead adopt a conservative alternative. AT finds the minimal number of steps to produce the object. We assume that every subobject, once available, can be used as often as needed to generate the object. A different approach would be to use Kolmogorov complexity^20,21^ applied to a given molecule, but this requires starting with a graphical representation, and a program to compute the graph of that molecule. The Kolmogorov complexity of a string is the shortest program that will output that string for a programming language capable of universal computation. This measure cannot be easily computed, because checking whether any single program will output the string is uncomputable, as it involves, at least, deciding whether the program stops. Running this program reflects nothing of the underlying process of how the molecule was constructed. Only late in the evolutionary process will molecules be produced by anything starting to resemble Turing machines, loops, stacks, tapes and so on^22^. Thus, using universal computation to assess molecules adds unrealistic dynamics, making the answer uncomputable. The assembly measure that we have presented here both uses realistic dynamics for molecules, using bonds as building blocks, and is computable for any molecule. The main work for detecting evolution and memory is done here by combining the assembly index and copy number of the objects.

The aim of AT is to develop a new understanding of the evolution of complex matter that naturally accounts for selection and history in terms of what operations are physically possible in constructing an object^23,24^. We will discuss AT as applied to chemical systems as the main application in this manuscript because their assembly index has been experimentally measured. For molecules, assembly index has a clear physical interpretation and has been validated as quantifying evidence of selection in its application to the detection of molecular signatures of life. However, we anticipate the theory to be sufficiently general to apply to a wide variety of other systems including polymers, cell morphology, graphs, images, computer programs, human languages and memes, as well as many others. The challenge in each case will be to construct an assembly space that has a clear physical meaning in terms of what operations can be caused to occur to make the object^23^ (Fig. 1).

In AT there are two important features of the context the object is found in. First, there must be objects in its environment that can constrain the steps to assemble the object and second these objects themselves have been selected because they must be retained over subsequent steps to physically instantiate the memory needed to build the target object. Among the most relatable examples are enzyme catalysts in biochemistry, which permit the formation of very unlikely molecules in large numbers because the enzymes themselves are also selected to exist with many copies. We make no distinction between the traditional notion of biological ‘individual’ and objects that are selected in the environment to quantify the selection necessary to produce a given configuration. Thus, our approach naturally accounts for well-known phenomena, such as niche construction, whereby organisms and environment are co-constructed and co-selected.

**Fig. 2:**
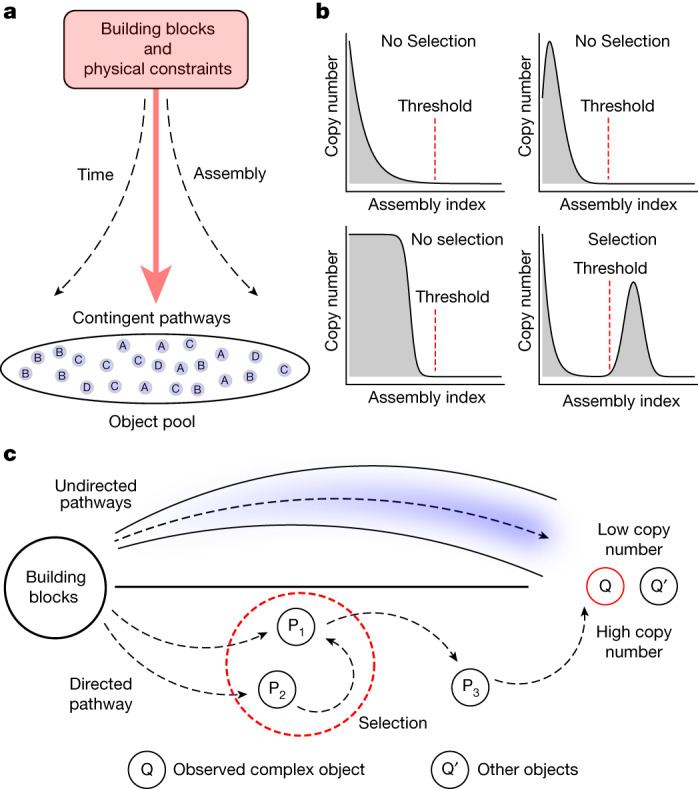
Selection in assembly space. **a**, Pictorial representation of the assembly space representing the formation of combinatorial object space from building blocks and physical constraints. **b**, Observed copy number distributions of objects at different assembly indices as an outcome of selection or no selection. **c**, Representation of physical pathways to construct objects with undirected and directed pathways (selected) leading to the low and high copy numbers of the observed object.

Copy number is important because a single example of a highly complex molecule (with a very high assembly index) could potentially be generated in a series of random events that become increasingly less likely with increasing assembly index. If we consider a forward-building assembly process (see Supplementary Information Sections 1 and 2 for details), without a specific target in mind, the number of possible objects that could be built at each recursive step grows super-exponentially in the absence of any constraints. The likelihood of finding and measuring more than one copy of an object therefore decreases super-exponentially with increasing assembly index in the absence of selection for a specified target. Objects with high assembly index, found in abundance, provide evidence of selection because of the combinatorially growing space of possible objects at each recursive assembly step (Fig. 2). Finding more than one identical copy indicates the presence of a non-random process generating the object.

## The assembly equation

We define assembly as the total amount of selection necessary to produce an ensemble of observed objects, quantified using equation (1):

1 $$A=\mathop{\sum }\limits_{i=1}^{N}{e}^{{a}_{i}}\left(\frac{{n}_{i}-1}{{N}_{{\rm{T}}}}\right)$$

where $A$ is the assembly of the ensemble, ${a}_{i}$ is the assembly index of object $i$, ${n}_{i}$ is its copy number, *N* is the total number of unique objects, *e* is Euler’s number and ${N}_{{\rm{T}}}$ is the total number of objects in the ensemble. Normalizing by the number of objects in the ensemble allows assembly to be compared between ensembles with different numbers of objects.

Assembly quantifies two competing effects, the difficulty of discovering new objects, but, once discovered, some objects become easier to make; this is indicative of how selection was required to discover and make them. The exponential growth of assembly with depth in assembly space, as quantified by assembly index, is derived by considering a linearly expanding assembly pool that has objects that combine at step $a\to a+1$, whereby an object at the assembly index $a$ combines with another object from the assembly pool. Discovering new objects at increasing depth in an assembly space gets increasingly harder with depth because the space of possibilities expands exponentially. Once the pathway for a new object has been discovered, the production of an object (copy number greater than 1) gets easier as the copy number increases because a high copy number implies that an object can be produced readily in a given context. Thus, the hardest innovation is making an object for the first time, which is equivalent to its discovery, followed by making the first copy of that object, but once an object exists in very high abundance it must already be relatively easy to make. Hence, assembly (*A*) scales linearly with copy number for more than one object for a fixed cost per object once a process has been discovered (see Supplementary Information Section 3 for additional details).

Increasing assembly ($A$) results from increasing copy numbers $n$ and increasing assembly indices $a$. If high values of assembly can be shown to capture cases in which selection has occurred, it implies that finding high assembly index objects in high abundance is a signature of selection. In AT, the information required at each step to construct the object is ‘stored’ within the object (Fig. 2). Each time two objects are combined from an assembly pool, the specificity of the combination process constitutes selection. As we will show, randomly combining objects within the assembly pool at each step does not constitute selection because no combinations exist in memory to be used again for building the same object. If, instead, certain combinations are preferentially used, it implies that a mechanism exists that selects the specific operations and, by extension, specific target objects to be generated. Later we will quantify the degree of selectivity by parameter $\alpha$ in the growth dynamics, which allows parameterizing selection in an empirically observable manner by parameterizing reuses of specific sets of operations (see Supplementary Information Section 3 for example).

Assembly as given in equation (1) is determined for identified finite and distinguishable objects (with copy number greater than 1) and their distinct assembly spaces. However, in real samples, there are almost always several different coexisting objects, which will include a common history for their formation. Transistors, for example, are used across several different technologies, suggesting a common subspace in the assembly spaces of many modern technologies that includes transistor-like objects. This common subspace, constituting the overlap in the assembly paths of distinct structures, is called a co-assembly space. By contrast, a joint assembly space of several objects is the combined assembly space required to generate those objects. As a potential extension of the assembly equation, to account for the joint assembly of objects, we expand the formulation of the assembly equation that includes the quantification of shared pathways to construct objects to determine the assembly ($A$) of an ensemble with different objects that share common history (Supplementary Information Section 3).

## Selection within assembly spaces

**Fig. 3:**
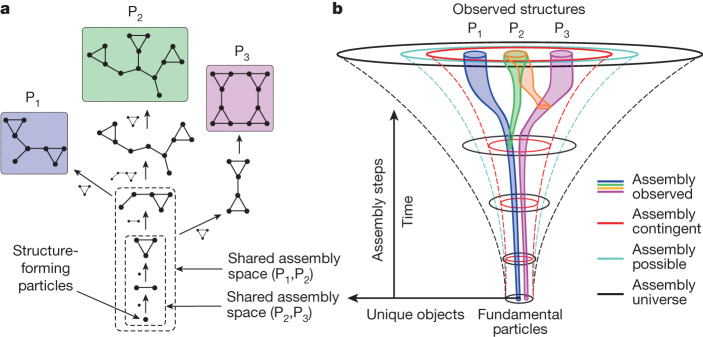
Assembly spaces. **a**, Assembly observed of the three objects shown as graphs (P_1_, P_2_ and P_3_) with their shared minimal construction process called their ‘joint assembly space’. **b**, Illustration of the expansion of the assembly universe, assembly possible, assembly contingent and assembly observed (see text for details). Assembly universe has no dynamics and is displayed with assembly steps as the time axis. Note that the figure illustrates their nested structure only, not the relative size of the spaces where each set is typically exponentially larger than the subset.

The concept of the assembly space allows us to understand how selection and historical contingency impose constraints on what can be made in the future. By aiming to detect ‘selection’, we mean a process similar to selection in Darwinian evolution. We do not, however, model functional differences that selection might act on. Instead, we account only for the specificity of selection—that some objects are more likely to be used to make new things and some are less likely. The only functionality we want to detect or describe is in the memory of the process to generate the object, with examples including a metabolic reaction network or a genome. This allows the three Lewontin conditions for evolution to hold^25^. A key feature of assembly spaces is that they are combinatorial, with objects combined at every step. Combinatorial spaces do not play a prominent role in current physics, because their objects are modelled as point particles and not as combinatorial objects (with limited exceptions). However, combinatorial objects are important in chemistry, biology and technology, in which most objects of interest (if not all) are hierarchical modular structures. More objects exist in assembly space than can be built in finite time with finite resources because the space of possibilities grows super-exponentially with the assembly index. To tame this explosive growth, in AT historical contingency is intrinsic with the space built compositionally, where items are combined recursively (accounting for hierarchical modularity) and this substantially constrains the number of possible objects. It is the combination of this compositionality with combinatorics that allows us to describe selection (Fig. 3).

To produce an assembly space, an observed object is broken down recursively to generate a set of elementary building units. These units can be used to then recursively construct the assembly pathways of the original object(s) to build what we call assembly observed, *A*_O_. *A*_O_ captures all histories for the construction of the observed object(s) from elementary building blocks, consistent with what physical operations are possible. Because objects in AT are compositional, they contain information about the larger space of possible objects from which they were selected. To see how, we first build an assembly space from the same building blocks in *A*_O_, which include all possible pathways for assembling any object composed of the same set elementary building blocks as our target object. The space so constructed is the assembly universe (*A*_U_).

In the assembly universe, all objects are possible with no rules, yielding a combinatorial explosion and with double exponential growth in the number of objects, as is characteristic of exploding state spaces and the adjacent possible (see Supplementary Information Section 4 for details). Although mathematically well defined, this double exponential growth is unphysical because the physical processes place restrictions on what is possible (in the case of molecules, an example is how quantum mechanics constrains the numbers of bonds per atom). The assembly universe also has no concept of directionality in time, as there is no ordering to construction processes. Because everything can exist, there is an implication that objects can be constructed independently of what has existed in the past and of resource or time constraints, which is not what we observe in the real universe. For most systems of interest, including in molecular assembly spaces, the number of molecules in the assembly universe is orders of magnitude larger than the amount of matter available in the cosmologically observable universe. There is no way to computationally build and exhaust the entire space, even for objects with relatively low assembly indices. For larger objects, such as proteins, this can be truly gigantic^26^. In AT, we do not observe all possible objects at a given depth in the assembly space because of selection, more reflective of what we see in the real universe. We next show how taking account of memory and resource limitation severely restricts the size of the space of what can be built, but also allows higher-assembly objects to be built before exhausting resources constructing all the possible lower-assembly objects. AT can account for selection precisely because of the historical contingency in the recursive construction of objects along assembly paths.

Assembly possible (*A*_P_) is the space of physically possible objects, which can be generated by means of the combinatorial expansion of all the known physical rules of object construction and allowing all rules to be available at every step to every object. This can be described by a dynamical model representing undirected forward dynamics in AT. When an object with assembly index $a$ combines with its own history, its assembly index increases by one, $a\to a+1$. If the resulting object can be made by means of other, shorter path(s), its assembly index will be smaller than $a+1$ or even $a$. Another assumption behind the dynamical model of undirected dynamics is a microscopically driven stochastic rule that uses existing objects uniformly: the probability of choosing an object with assembly index $a$ to be combined with any other object is proportional to ${N}_{a}$, the number of objects with assembly index $a$ (see Supplementary Information Section 5 for further details).

Within assembly possible, assembly contingent (*A*_C_) describes the possible space of objects where history, and selection on that history, matter. Historical contingency is introduced by assuming that only the knowledge or constraints built on a given path can be used in the future, or with different paths interacting in cases in which selected objects that had not interacted previously now interact. We define the probability ${P}_{a}$ of an object being selected with assembly index ($a$) as ${P}_{a}\propto {({N}_{a})}^{\alpha }$, where ${N}_{a}$ is the number of objects with assembly index $a$. Here, $\alpha$ parameterizes the degree of selection: for $\alpha =1$ all objects that have been assembled in the past are available for reuse, and for $0\le \alpha  < 1$, only a subset (that grows non-linearly with assembly index) are available for reuse, indicating that selection has occurred. This leads to the growth dynamics:

2 $$\frac{{\rm{d}}{N}_{a+1}}{{\rm{d}}t}={k}_{{\rm{d}}}{({N}_{a})}^{\alpha }$$

where ${k}_{{\rm{d}}}$ represents the rate of discovery (expansion rate) of new objects. For $\alpha =1$, there is historical dependence without selection. We build assembly paths by taking two randomly chosen objects from the assembly pool and combining them; if a new object is formed, it is added back into the pool. Here we are building random objects, but these are fundamentally different from random combinatorial objects because the randomness we implement is distributed across the recursive construction steps leading to an object (see Supplementary Information Section 5 for solutions). The case of $\alpha =1$, in which there is historical dependence but no selection, defines the boundary of assembly possible.

**Fig. 4:**
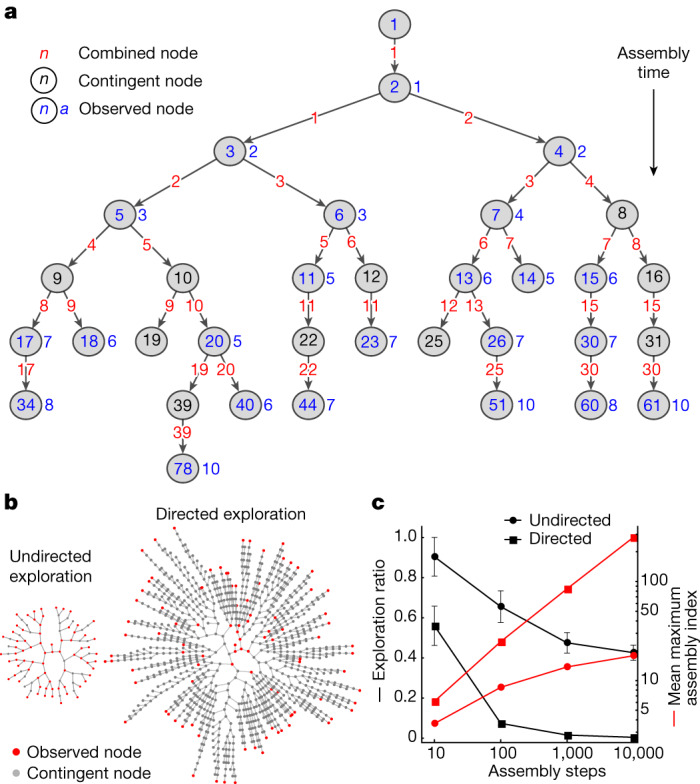
Undirected and directed exploration in a forward assembly process. **a**, The joint assembly space of polymeric chains (with their lengths indicated) after 30 steps created by combining randomly selected polymers from the assembly pool. The length of the realized polymers is shown in blue (observed nodes), whereas nodes shown in black represent polymers that have not been realized but are part of the joint assembly space of all realized objects (contingent nodes). For simplicity of representing the joint assembly space, the edge nodes (shown in red) represent the combined node along the directed graph. **b**, The comparison between undirected and directed exploration after 100 assembly steps using a graph with radial embedding (observed and contingent nodes shown in red and grey, respectively). **c**, The mean and standard deviation of the exploration ratio (defined by the ratio of the number of observed nodes and the number of total nodes, which includes observed and contingent nodes) and mean maximum assembly index. *n* is 25 runs all averaged up to 10^4^ assembly steps.

Within assembly possible, the assembly contingent (*A*_C_) is the space of possible configurations of objects where $0\le \alpha  < 1$, that is, where selection is possible, and the objects found in the space are controlled by a path-dependency contingent on each object that has already been built. The growth of the assembly contingent is much slower than exponential; indeed, not all possible paths are explored equally. Instead, the dynamics are channelled by constraints imposed by the selectivity emerging along specific paths. Indeed, a signature of selection in assembly spaces is a slower-than-exponential growth of the number of unique objects. To show this, we use a simple phenomenological model of linear polymers to demonstrate how assembly differentiates cases when selection happens. Starting with a single monomer in the assembly pool, the undirected exploration process combines two randomly selected polymers and adds them back to the assembly pool. In the case of directed exploration with selection, the polymer that has been created most recently is selected to join a randomly selected polymer from the assembly pool. For both directed and undirected exploration, this process was iterated up to 10^4^ steps and repeated 25 times. For each observed polymer in the assembly pool, the shortest pathway was generated. For each run, the assembly space of multiple coexisting polymers, their joint assembly space, was approximated by the union of the shortest pathways of all observed polymers. An example of joint assembly space in an undirected exploration up to 30 steps is shown in Fig. 4a.

Comparison between the explored joint assembly space in undirected and directed exploration up to 100 steps is shown in Fig. 4b (see Supplementary Information Section 6 for details). To quantify the degree of exploration at a given assembly step, we calculated the exploration ratio, defined by the ratio of observed nodes to total number of nodes present in the joint assembly space. Figure 4c shows the exploration ratio and the mean maximum assembly index observed, approximated by ${\log }_{2}\left(n\right)$, where $n$ is the length of the polymer for the undirected and directed exploration processes (both upper and lower bounds scale as ${\log }_{2}\left(n\right)$ in leading order). Here, the mean maximum assembly index was estimated by calculating the assembly index of the mean value of the longest observed polymeric chains over 25 runs. Comparing the directed process to the undirected exploration illustrates a central principle: the signal of selection is simply a lower exploration ratio and higher complexity (as defined by the maximum assembly index). The observation of a lower exploration ratio in the directed process than in the undirected process is the evidence of the presence of selectivity in the combination process between the polymers existing in the assembly pool. The process representing sorting and selecting chains within the assembly pool represents an outcome of a physical process leading to selection (see Supplementary Information Section 7 for an additional model).

We conjecture that, the ‘more assembled’ an ensemble of objects, the more selection is required for it to come into existence. The historical contingency in AT means that assembly dynamics explores higher-assembly objects before exhausting all lower-assembly objects, leading to a vast separation in scales separating the number of objects that could have been explored versus those that are actually constructed following a particular path. For example, proteins built both from d and l amino acids and their pathways are part of assembly possible, but, within an assembly contingent trajectory, only proteins constructed out of l amino acids might be present, because of early selection events. This early symmetry breaking along historically contingent paths is a fundamental property of all assembly processes. It introduces an ‘assembly time’ that ticks at each object being made: assembly physics includes an explicit arrow of time intrinsic to the structure of objects.

## Assembly unifies selection with physics

**Fig. 5:**
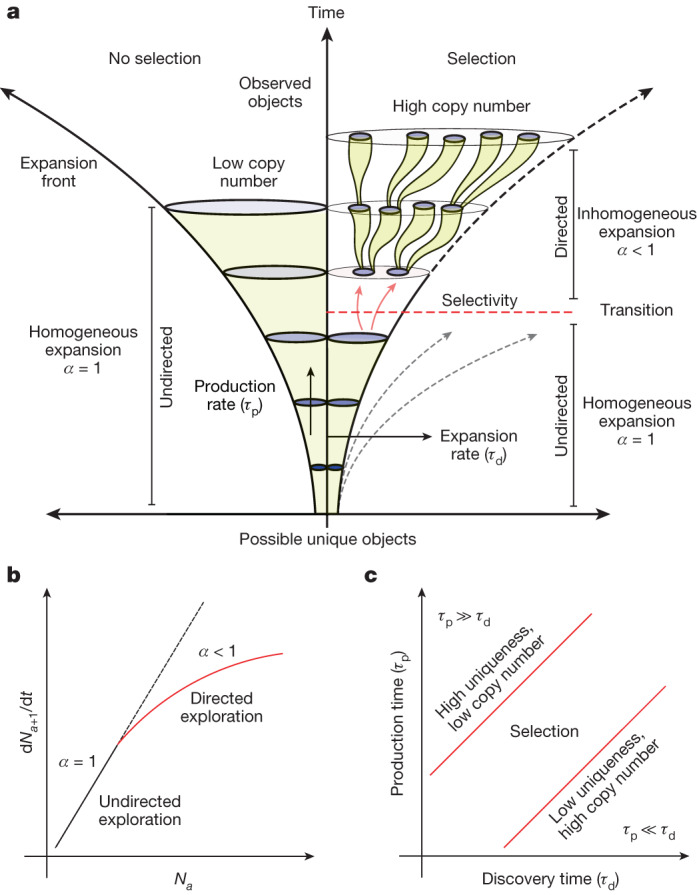
Selection and evolution in assembly space. **a**, Assembly processes with and without selection. The selection process is defined by a transition from undirected to directed exploration. The parameter $\alpha$ represents the selectivity of the assembly process ($\alpha =1$: undirected/random expansion, $\alpha  < 1$: directed expansion). Undirected exploration leads to the fast homogeneous expansion of discovered objects in the assembly space, whereas directed exploration leads to a process that is more like a depth-first search. Here, ${\tau }_{{\rm{d}}}$ is the characteristic timescale of discovery, determining the growth of the expansion front, and ${\tau }_{{\rm{p}}}$ is the characteristic timescale of production that determines the rate of formation of objects (increasing copy number). **b**, Rate of discovery of unique objects at assembly $a+1$ versus number of objects at assembly $a$. The transition of $\alpha =1$ to $\alpha  < 1$ represents the emergence of selectivity limiting the discovery of new objects. **c**, Phase space defined by the production (${\tau }_{{\rm{p}}}$) and discovery (${\tau }_{{\rm{d}}}$) timescales. The figure shows three different regimes: (1) ${\tau }_{{\rm{d}}}\ll {\tau }_{{\rm{p}}}$, (2) ${\tau }_{{\rm{d}}}\gg {\tau }_{{\rm{p}}}$, and (3) ${\tau }_{{\rm{d}}}\approx {\tau }_{{\rm{p}}}$. Selection is unlikely to emerge in regimes 1 and 2, and is possible in regime 3.

In the real universe, objects can be built only from parts that already exist. The discovery of new objects is therefore historically contingent. The rate of discovery of new objects can be defined by the expansion rate (${k}_{{\rm{d}}}$) from equation (2), introducing a characteristic timescale ${\tau }_{{\rm{d}}}\approx \frac{1}{{k}_{{\rm{d}}}}$, defined as the discovery time. In addition, once a pathway to build an object is discovered, the object can be reproduced if the mechanism in its environment is selected to build it again. Thus far, we have considered discovery dynamics within the assembly spaces and did not account for the abundance or copy number of the observed objects when discovered. To include copy number in the dynamics of AT, we must introduce a second timescale, the rate of production (${k}_{{\rm{p}}}$) of a specific object, with a characteristic production timescale ${\tau }_{{\rm{p}}}\approx \frac{1}{{k}_{{\rm{p}}}}$ (Fig. 5). For simplicity, we assume that selectivity and interaction among emerging objects are similar across assembled objects. Defining these two distinct timescales for initial discovery of an object and making copies of existing objects allows us to determine the regimes in which selection is possible (Fig. 5).

For $\frac{{\tau }_{{\rm{p}}}}{{\tau }_{{\rm{d}}}}\gg 1$, whereby objects are discovered quickly but reproduced slowly, the expansion of assembly space is too fast under mass constraints to accumulate a high abundance of any distinguishable objects, leading to a combinatorial explosion of unique objects with low copy numbers. This is consistent with how some unconstrained prebiotic synthesis reactions, such as the formose reaction, end up producing tar, which is composed of a large number of molecules with too low a copy number to be individually identifiable^27,28^. Selection and evolution cannot emerge if new objects are generated on timescales so fast that resources are not available for making more copies of those objects that already exist. For $\frac{{\tau }_{{\rm{p}}}}{{\tau }_{{\rm{d}}}}\ll 1$, objects are reproduced quickly but new ones are discovered slowly. Here resources are primarily consumed in producing additional copies of objects that already exist. Typically, new objects are discovered infrequently. This leads to a high abundance of objects produced by extreme constraints, which could limit the further growth of assembly space. This illustrates how exploration versus exploitation can play out in AT. Significant separation of the two timescales of discovery of new objects and (re)production of selected objects results in either a combinatorial explosion of objects with low copy numbers or, conversely, high copy numbers of low assembly objects. In both cases, we will not observe trajectories that grow more complex structures.

The emergence of selection and open-ended evolution in a physical system should occur in the transition regime where there is only a small separation in the timescales between discovering new objects and reproducing ones that are selected, for example the region located between ${\tau }_{{\rm{d}}}\ll {\tau }_{{\rm{p}}}$ and ${\tau }_{{\rm{d}}}\gg {\tau }_{{\rm{p}}}$ (Fig. 5). To investigate discovery and production dynamics simultaneously, we introduce mass action kinetics in the framework of AT. Our aim is to demonstrate how the generation of novelty can be described alongside selection in a forward process (thus unifying key features of life with physics) and how measuring assembly identifies how much selection occurred. We do so by studying phenomenological models, with the understanding that we are putting selection in by hand in our examples to demonstrate foundational principles of how assembly quantifies selection. To explore this, we consider a forward assembly process whereby the copy numbers of emerging objects follow homogeneous kinetics, together with the discovery dynamics as given by equation (2). With the discovery of new unique objects over time, symmetry breaking in the construction of contingent assembly paths will create a network of growing branches within the assembly possible. In principle, interactions among existing objects and external factors lead to discovery of new objects, expanding the space of possible future objects. Such events can drastically change the copy number distribution of objects at various assembly indices, depending on the emerging kinetics in the formation of new objects. By combining discovery and production kinetics in a simplified formulation, we estimate copy numbers of objects at different assembly indices and show assembly of the ensemble over time in the forward process at different degrees of selection (see Supplementary Information Section 8 for an example).

The interplay between the two characteristic timescales describes how discovery dynamics (${{\tau }_{{\rm{d}}}\approx 1/k}_{{\rm{d}}}$) and forward kinetics (${\tau }_{{\rm{p}}}\approx 1/{k}_{{\rm{p}}}$), together with selection (characterized by the selection parameter $\alpha$), are essential for driving processes towards creating higher-assembly objects. This is characteristic of trajectories within assembly contingent. Assembly captures key features of how the open-ended growth of complexity can occur within a restricted space only by generating new objects with increasing assembly indices, while also producing them with a high copy number. Selectivity ($\alpha  < 1$) together with comparable production timescales (${\tau }_{{\rm{d}}}\approx {\tau }_{{\rm{p}}}$) is essential for the production of high assembly ensembles. This suggests that selectivity in an unknown physical process can be explained by experimentally detecting the number of objects, their assembly index and copy number as a function of time. Considering molecules as objects and assuming that molecules observed using analytical techniques such as mass spectrometry implies a high copy number, the discovery rate and the selection index ($\alpha$) can be computed from the temporal data of observed molecules at all assembly indices.

## Conclusions

We have introduced the foundations of AT and how it can be implemented to quantify the degree of selection found in an ensemble of evolved objects, agnostic to the detailed formation mechanisms of the objects or knowing a priori which objects are products of units of selection. To do so, we introduced a quantity, assembly, built from two quantities: the number of copies of an object and its assembly index, where the assembly index is the minimal number of recursive steps necessary to build the object (its size). We demonstrated how AT allows a unified language for describing selection and the generation of novelty by showing how it quantifies the discovery and production of selected objects in a forward process described by mass action kinetics. AT provides a framework to unify descriptions of selection across physics and biology, with the potential to build a new physics that emerges in chemistry in which history and causal contingency through selection must start to play a prominent role in our descriptions of matter. For molecules, computing the assembly index is not explicitly necessary, because the assembly index can be probed directly experimentally with high accuracy with spectroscopy techniques including mass spectroscopy, infrared and nuclear magnetic resonance spectroscopy^29^.

## Methods

All the calculations were performed using Mathematica 13 (Wolfram Ltd). In addition, assembly index calculations on polymeric strings in the Supplementary Information were performed using a string assembly calculator previously developed using Python and C++.

### Reporting summary

Further information on research design is available in the Nature Portfolio Reporting Summary linked to this article.

## Online content

Any methods, additional references, Nature Portfolio reporting summaries, source data, extended data, supplementary information, acknowledgements, peer review information; details of author contributions and competing interests; and statements of data and code availability are available at 10.1038/s41586-023-06600-9.

### Supplementary information

Supplementary Information Details of the mathematical models, simulations and examples used in the manuscript, Supplementary Sections 1–10, Figs. 1–18 and one additional reference.

Reporting Summary

## Data Availability

All Mathematica Notebooks used to perform the calculations are available at https://github.com/croningp/assemblyphysics. The string assembly calculator and the dataset of assembly index calculations is available from the Zenodo repository 10.5281/zenodo.8017327.
