# Supplementary material for: Assembly theory explains and quantifies selection and evolution
Source: Nature. 2023 Oct 4;622(7982):321–8. doi: 10.1038/s41586-023-06600-9 (PMC10567559; doi:10.1038/s41586-023-06600-9)
Supplement: Supplementary file 1 — Details of the mathematical models, simulations and examples used in the manuscript, Supplementary Sections 1–10, Figs. 1–18 and one additional reference. [file 41586_2023_6600_MOESM1_ESM.pdf]

nature portfolio

https://doi.org/10.1038/s41586-023-06600-9

# Supplementary information

# Assembly theory explains and quantifies selection and evolution

In the format provided by the authors and unedited

# Supplementary Information

## Assembly Theory Explains and Quantifies Selection and Evolution

Abhishek Sharma,[1†] Dániel Czégel,[2,3†] Michael Lachmann,[4] Christopher P. Kempes,[4] Sara I. Walker,[2,5]* Leroy Cronin[1]*

† Equal contribution [1] School of Chemistry, University of Glasgow, Glasgow, G12 8QQ, UK.

[2] BEYOND Center for Fundamental Concepts in Science, Arizona State University, Tempe, AZ, USA

[3] Institute of Evolution, Centre for Ecological Research, Budapest, Hungary

[4] The Santa Fe Institute, Santa Fe, New Mexico, USA

[5] School of Earth and Space Exploration, Arizona State University, Tempe, AZ, USA

*Corresponding authors' emails: Lee.Cronin@glasgow.ac.uk, sara.i.walker@asu.edu

## Table of Contents

1. Quantifying Assembly Paths in Expanding Linear Space ... 3
2. Estimating the shortest assembly path for a linear chain ... 5
3. Quantifying Assembly of Ensemble ... 9
3.1 Exponential Dependence on Assembly Index ... 9
3.2 Linear dependence on copy number ... 11
3.3 Assembly in Joint Assembly Space ... 12
4. Assembly Universe and super-exponential expansion ... 15
4.1 Scaling in assembly universe ... 15
5. Assembly Possible and Assembly Contingent ... 17
5.1 Dynamics of Assembly Possible ... 17
5.2 Dynamics of Assembly Contingent ... 18
6. Joint Assembly Space in Linear Chain Model ... 20
6.1 Joint Assembly Space of observed objects ... 20
6.2 Joint Assembly Space in the forward process ... 21
7. Distribution of unique objects in a forward process with different selectivity ... 24
8. Assembly Contingent combined with mass transfer kinetics ... 26
9. Code Availability ... 34
10. References ... 34

# 1. Quantifying Assembly Paths in Expanding Linear Space

The Assembly pool at a given step is a set of possible structures that have been formed previously along an assembly path and are accessible at the next assembly step to create higher-order structures. To illustrate this process, here we generate a complete combinatorial assembly pool for linear chains defined as integers which are equivalent to linear polymers constructed from a single monomeric unit. To minimize the cost of enumeration, at each step of the forward process, we consider all possible combinations between the objects created at the last step with all the objects present in the assembly pool.

As an example, the assembly pool after three full combinatorial steps is given by,

$P = \{1, 2, 3, 4, 5, 6, 7, 8\}$

with connections based on joining operations are given by,

$C = \{\{1 \rightarrow 2\}, \{2 \rightarrow 3, 2 \rightarrow 4\}, \{3 \rightarrow 4, 3 \rightarrow 5, 3 \rightarrow 6, 3 \rightarrow 7, 4 \rightarrow 5, 4 \rightarrow 6, 4 \rightarrow 7, 4 \rightarrow 8\}\}$.

It is important to note that these combination steps do not necessarily correspond to the assembly index which represents the shortest path to create an object. In general, within the assembly pool generated by forward steps, any object will only appear as an outcome of the assembly process (combining integers) until the slowest path to form the object has been achieved. The slowest step is the one where at each step, one fundamental unit adds to the current structure along the assembly path. For example, in a linear chain, the slowest path to create a chain length 3 is $1 \rightarrow 2 \rightarrow 3$, which includes two steps. The complete assembly pool at the end of the two steps is $\{\{1\}, \{2\}, \{3,4\}\}$ which is formed from the combinations $\{1, 2, \{1 + 2, 2 + 2\}\}$. Here, we also assume parallel concurrent processes exist such that $\{3,4\}$ both coexist at the end of two steps. Considering the third step, by enumerating all the combinations between the integer $\{3\}$ and $\{1, 2, 3, 4\}$, the new unique chains that will be added to the assembly pool are $\{4, 5, 6, 7\}$ and the formation chain length 3 is not possible as a new forward step. In a similar way, for integer $\{4\}$, the new unique chains in the next forward step will be $\{5, 6, 7, 8\}$. The fastest-growing assembly path in the assembly pool is when at each step the highest assembly object combines with itself, for example in a linear chain the fastest-growing path is $1 \rightarrow 2 \rightarrow 4 \rightarrow 8 \rightarrow 16$. So, starting with a chain length 1 as the fundamental object, after $n$ steps, the shortest and the longest chain lengths are $n + 1$ and $2^n$, respectively. As an example, Fig. S1 shows the fully combinatorial assembly pool after four steps with the shortest and longest paths highlighted in red and blue, and examples for chain lengths 6 and 7.

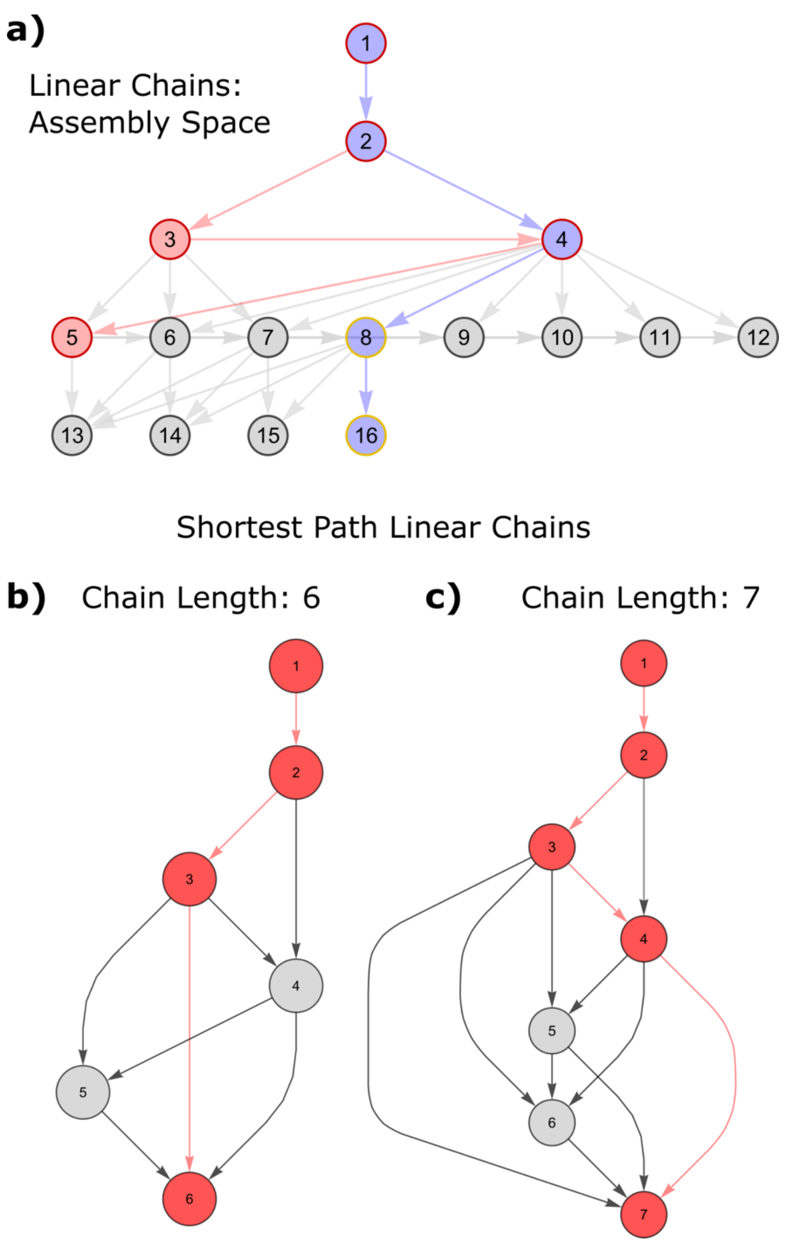


**Fig. S1 Assembly Space for linear chains.** (a) The figure shows the assembly space of one-dimensional linear chains up to four assembly steps. The path in red (up to 5) and blue (up to 16) shows the slowest and fastest assembly steps. (b, c) Figure shows the shortest path for linear chains of lengths 6 and 7. Note that assembly index which represents the shortest path in a serial process for chain length 6 and 7 is 3 and 4 respectively.

In another way, the slowest path to create a chain with length $n$, starting with the fundamental object with length 1 (single monomer), consists of $n-1$ steps. Hence, enumerating all the possible paths to create a chain of length $n$, requires enumeration of the combinatorial space up to $n-1$ steps only. All the chains with length < $n$, will contribute to the possible assembly paths to create a chain of length $n$. The longest chain length possible in a similar number of steps is $2^{n-1}$. Hence, in a fully combinatorial assembly space, to create a chain length $n$ with all possible pathways, the number of additional chains which can potentially form during the combination steps is $2^{n-1}-n$. This can be approximated as $2^{n-1}$ which indicates that the potential additional chains increase exponentially with

$n$. This shows that in the absence of selection, the combinatorial space expands exponentially for integers. For example, Fig. S2 (a-c) shows combinatorial assembly space for chain lengths 4, 5, and 6 together with additional chains which can also be formed in $n-1$ steps.

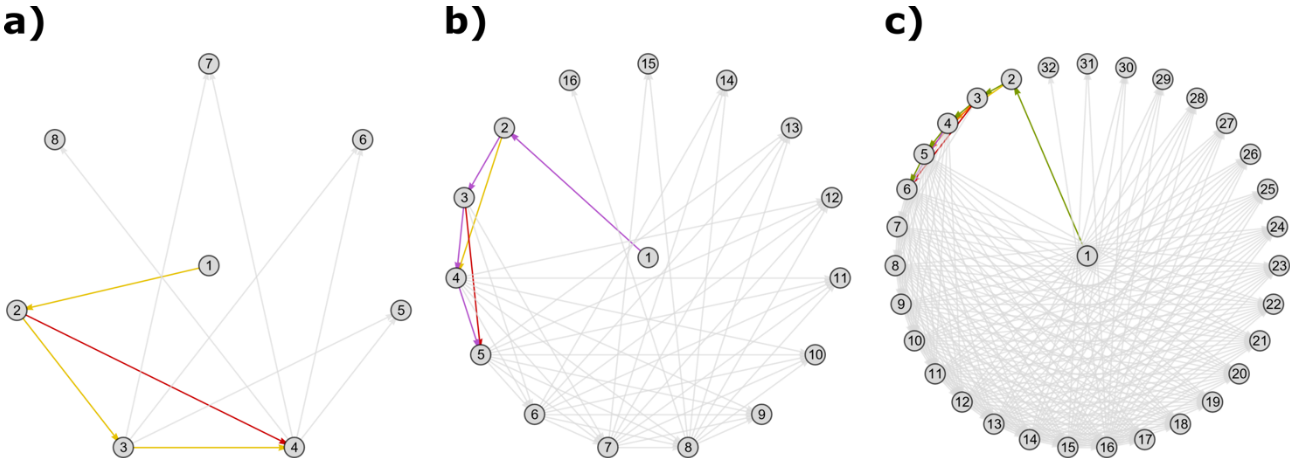


**Fig. S2 Combinatorial assembly space of linear chains.** (a-c) shows combinatorial assembly space for chain lengths 4, 5, and 6. The coloured paths represent potential assembly paths to reach the specific chain length and paths in the grey lead to the formation of additional chains. For chain lengths 4, 5, and 6, the number of additional chains formed is $2^3-4=4$, $2^4-5=11$, and $2^5-6=26$ respectively.

## 2. Estimating the shortest assembly path for a linear chain

For a linear chain of length $n$, the assembly index (number of steps required by the shortest path to construct the chain) which quantifies that both upper and lower bounds scales as $\log_2(n)$ in the leading order. The exact assembly index can be estimated by generating the pathway and counting the number of steps. At various calculations, we approximate assembly index as $a \sim \log_2(n)$ for simplicity. As an example, for a simple chain, the assembly path to construct a chain of length 7 is $1 \rightarrow 2 \rightarrow 3 \rightarrow 4 \rightarrow 7$, hence the assembly index is 4. Similarly, the pathway to construct a chain of length 8 is given by $1 \rightarrow 2 \rightarrow 4 \rightarrow 8$ and hence its assembly index is 3. The scaling of the assembly index at the leading order with chain length is shown in Fig. S3.

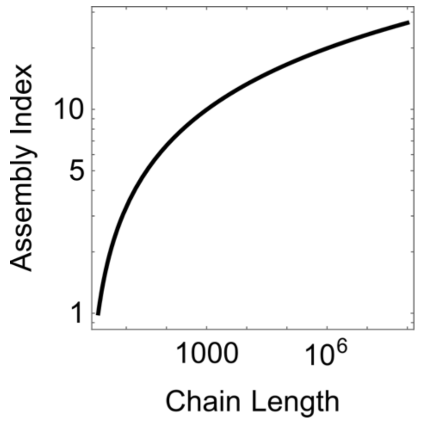


**Fig. S3 Assembly Index of linear chains.** The figure shows the assembly index of linear chains *vs.* chain length estimated using $a \sim \log_2(n)$ where $n$ is the chain length.

To estimate the potential pathways and search the shortest path to the assembly of a chain of length $n$, first, the assembly pool is generated up to $n-1$ steps such that slowest step can be included. All the chains in the assembly pool with lengths longer than the given chain length can be excluded. For example, considering chain length 5, the generated combinatorial assembly pool is shown in Fig. S2 (b) by enumerating combinations up to 4 steps. The example pathways for chain lengths 4, 5, and 6 are given below.

Example pathways for chain length 5:

$\{1 \rightarrow 2 \rightarrow 3 \rightarrow 4 \rightarrow 5\}$,

$\{1 \rightarrow 2 \rightarrow 4 \rightarrow 5\}$,

$\{1 \rightarrow 2 \rightarrow 3 \rightarrow 5\}$,

Example pathways for chain length 6:

$\{1 \rightarrow 2 \rightarrow 3 \rightarrow 4 \rightarrow 5 \rightarrow 6\}$,

$\{1 \rightarrow 2 \rightarrow 3 \rightarrow 4 \rightarrow 6\}$,

$\{1 \rightarrow 2 \rightarrow 3 \rightarrow 5 \rightarrow 6\}$,

$\{1 \rightarrow 2 \rightarrow 3 \rightarrow 6\}$,

$\{1 \rightarrow 2 \rightarrow 4 \rightarrow 5 \rightarrow 6\}$

$\{1 \rightarrow 2 \rightarrow 4 \rightarrow 6\}$

Example pathways for chain length 7:

$\{1 \rightarrow 2 \rightarrow 3 \rightarrow 4 \rightarrow 7\}$,

$\{1 \rightarrow 2 \rightarrow 3 \rightarrow 5 \rightarrow 7\}$,

$\{1 \rightarrow 2 \rightarrow 3 \rightarrow 5 \rightarrow 6 \rightarrow 7\}$,

$\{1 \rightarrow 2 \rightarrow 4 \rightarrow 5 \rightarrow 7\}$,

$\{1 \rightarrow 2 \rightarrow 4 \rightarrow 6 \rightarrow 7\}$,

$\{1 \rightarrow 2 \rightarrow 3 \rightarrow 6 \rightarrow 7\}$,

$\{1 \rightarrow 2 \rightarrow 3 \rightarrow 4 \rightarrow 6 \rightarrow 7\}$,

$\{1 \rightarrow 2 \rightarrow 3 \rightarrow 4 \rightarrow 5 \rightarrow 6 \rightarrow 7\}$

While creating all the possible paths, as explained previously, the number of additional possible polymeric chains which can be created while assembling a chain length $n$ are given by $2^{n-1} - n$, see Fig. S4.

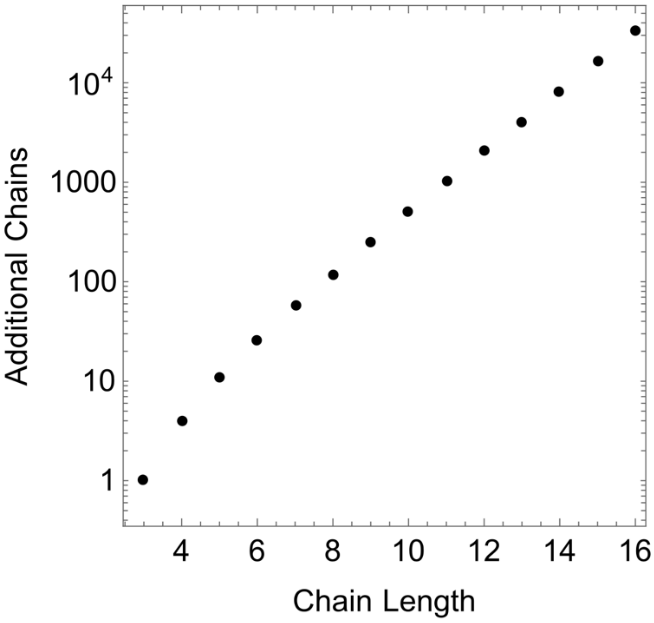


**Fig. S4 Enumeration of the additionally generated chains up to the longest path at different chain lengths.** The number of additional chains is given by $(2^{n-1} - n)$.

To calculate the shortest path for a given chain length $n$, we start with chain length $n$ and split it into sub-chains depending on if $n$ is odd or even. If $n$ is even, the sub-chain is $n/2$ and if $n$ is odd, sub-chains are $\{\lfloor n/2 \rfloor, \lceil n/2 \rceil\}$. This splitting operation is performed recursively for $p$ times, where $p$ is given by $\lceil \log_2(n) \rceil$, where the final sub-chains will be the fundamental chains of length 1. Using the sub-chains, the assembly pathway can be generated.

As an example, to generate the shortest path for chain length 7 (assembly index 4) as described above the set of sub-chains created is given by $\Big\{\{7\}, \{3,4\}, \big\{\{1,2\}, \{2\}\big\}, \{1\}\Big\}$. Hence, the shortest pathway to create chain length 7 involve $\{1,2,3,4,7\}$, hence the assembly index can be estimated by counting the number of edges in the graph representing the set of sub-chains. It is important to note that for a simple system like linear chains, there is more than one possible shortest pathway, and the current scheme only quantifies one possible shortest pathway which is used to describe here as the assembly path. For more complex and heterogeneous systems like molecules, the shortest path becomes more unique.

The combinatorial assembly space for linear chains formed from one monomeric unit is exponential at the leading order with respect to the assembly index. As described previously, for a linear chain of

length $n$, the assembly index at the leading order can be approximated by $a \sim \log_2(n)$. So, at each assembly step $(a)$ there are approximately $2^a$ possible chains. To keep the terminology clear, assembly index represents the minimum number of steps to create an object in a serial process, however here assembly steps represent combinatorial process which also includes concurrent steps.

Additionally, if we introduce two (A, B) or three monomers (A, B, C), the fully combinatorial assembly space grows faster as various configurations of polymer sequences could have the same assembly index. To enumerate all the polymeric sequences up to a given step $a$, the total number of $2^a$ combinatorial steps are required. We used the String Assembly Calculator[1] to calculate the assembly index of all possible polymeric sequences with two monomers up to length 16. The total number of possible polymeric sequences using two monomers A and B created up to length 16 is given by geometric sum $2\left(\frac{2^{16}-1}{2-1}\right) = 131{,}070$ where all the sequences were considered distinct. Similarly, the total number of sequences up to three monomeric units up to length 16 is $3\left(\frac{3^{16}-1}{3-1}\right) = 64{,}570{,}080$. We generated all these polymeric sequences using a Python code and calculated the assembly index using the String Assembly Calculator which enumerates all combinations up length 16. The distribution of polymeric sequences with respect to assembly indices is shown in Fig. S5.

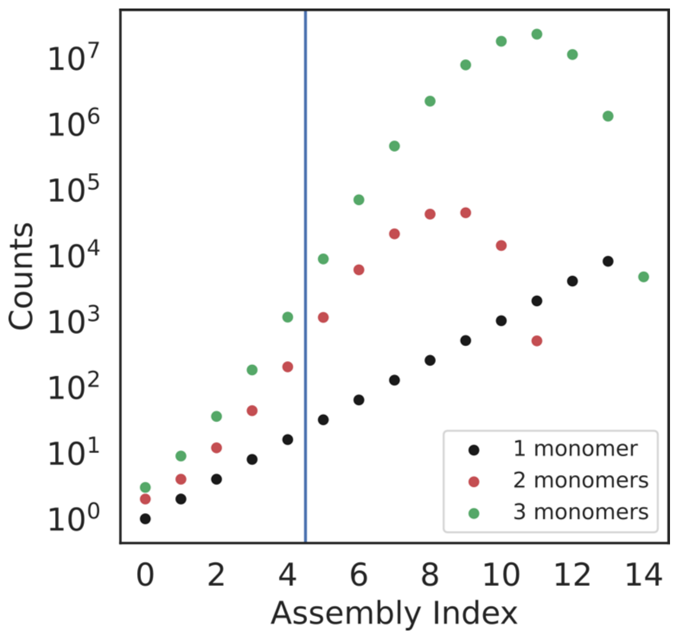


**Fig. S5 Enumerating possible structures in polymer space with one, two, and three monomers.** The figure shows the number of structures in the fully combinatorial assembly space *vs.* assembly index for polymeric sequences with one, two, and three monomeric units. The data points to the left of the blue line up to assembly index 4 show complete enumeration and on the right show partial enumeration.

## 3. Quantifying Assembly of an Ensemble

### 3.1 Exponential Dependence on Assembly Index

The assembly pool constitutes the number of unique objects that emerged from the contingent history and can be used further along the assembly path. For a single isolated chain, at assembly step $a \rightarrow a+1$, the set of objects available from the contingent history is $\{p_0, p_1, p_2, p_3 \dots p_a\}$, which increases linearly with the number of assembly steps. Here, $p_0$ represent the fundamental particle or building block. When a limited number of multiple objects coexist and have been observed/measured, in principle, the assembly pool of the joint assembly space also expands linearly in the forward process. Also, in the case of two shared assembly pathways, the number of objects in the assembly pool is given by $\{p_0, p_1, p_2, p_3, p_4, p_5 \dots p_i, q_6, q_7, q_8 \dots q_j\}$, assuming $q_6$ is formed from $p_5$, and $\{p_1, p_2, p_3, p_4, p_5\}$ constitutes objects in the shared assembly space, see Fig. S6.

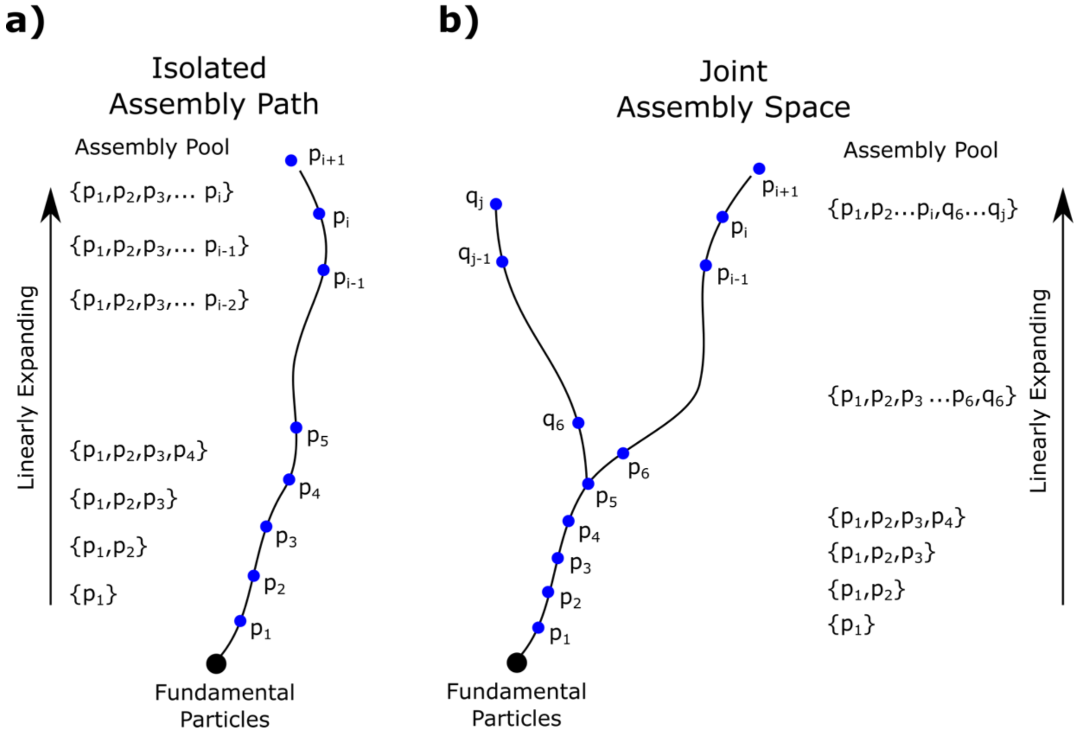


**Fig. S6 Growth of Assembly Pool with assembly steps.** (a) An isolated assembly path up $p_{i+1}$ contingent nodes. (b) Joint Assembly space with two branches up to $p_{i+1}$ and $q_j$, splitting at $p_5$, $p_5 \rightarrow p_6$ and $p_5 \rightarrow q_6$. In both cases, the assembly pool expands linearly (note that in the figure, fundamental particles are not shown in the assembly pool however they also constitute the assembly pool).

In this case, as well, the assembly pool increases faster than the single isolated chain, however, the expansion is still linear. The linear expansion of the assembly pool along an isolated assembly path, or in a joint assembly space splitting into two assembly paths, is shown in Fig. S6. In the case of the joint assembly space, the larger the shared assembly paths of the observed objects, the smaller the growth rate of the expansion of the assembly pool. In the extreme case which is unlikely to be

observed in most physical systems, where each object along the assembly path is observed and generates $k$ unique objects at each assembly step, in that case, the assembly pool (excluding the fundamental particles) available at step $a \to a + 1$ is given by the summation of a geometric sequence $\frac{k^a - 1}{k-1}$, where $k$ is the number of unique objects formed at the $(a + 1)^{\text{th}}$ step from each object at the $a^{\text{th}}$ step.

While considering a linearly expanding assembly pool where at step $a \to a + 1$, when the object at assembly index $a$ combines with another object from the assembly pool, we excluded the internal structure of the objects for the combination operation. When two objects from the assembly pool combine along the assembly path, the combination process comprises two aspects: *object selection* and *object construction*. O*bject selection* determines which two objects are to be combined at a given assembly step along the assembly path, and *object construction* defines how two selected objects are combined.

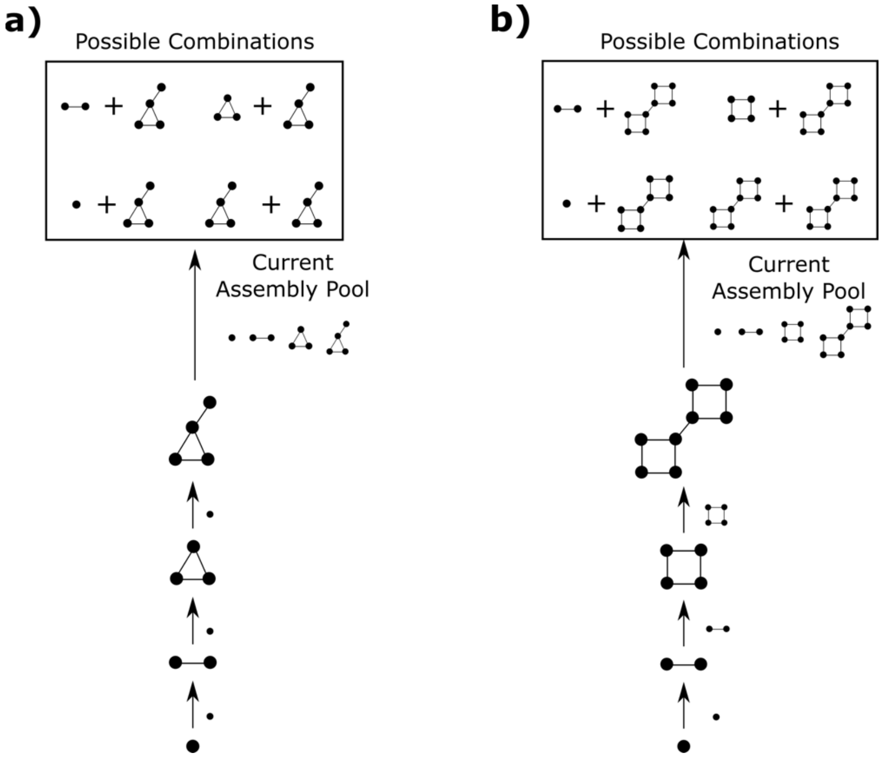


**Fig. S7 Combinatorial Assembly Space with linearly increasing Assembly Pool.** (a) An isolated assembly path where at each assembly step, the structure from the previous assembly step combines with a single fundamental particle. (b) An isolated assembly path where at each assembly step, the structure from the previous assembly step combines with itself. In both cases, after three steps, the potential pairwise combinations from the objects present in the assembly pool are shown. In both cases, we assume the building blocks (fundamental particles) are the point particles.

Consider the simplest case of a one-dimensional polymeric string of length $n$ that needs to be connected to a single monomer. If there is a constraint of a single bond, there are $n$ possible ways to connect the string with the monomer. In the absence of any bond constraints, there are $2^n - 1$ ways

to connect the monomer to the polymeric string. The combinatorial space for *object construction* for two selected objects will expand much faster when two extended objects such as two polymeric units are present instead of a monomeric unit. Fig. S7 shows the potential combinatorial space for object construction in a slow and fast-growing assembly space. Hence, even with linearly expanding assembly pool, the number of potential combinations of new objects at any assembly index considering object *selection* and *construction* is at least exponential. This suggests that the contribution of the construction process to the contingent power of an observed object must have at least exponential dependence on the assembly index $A \propto e^{f(a_i)}$.

### 3.2 Linear dependence on copy number

In the assembly process, there are two distinct characteristic time scales which govern the dynamics of the discovery of unique objects, and their copy numbers. These time scales are defined as discovery time scale ($\tau_\mathrm{d}$) and production timescale ($\tau_\mathrm{p}$). The characteristic timescale $\tau_\mathrm{d}$ of the discovery dynamics represents the time scale required by the assembly process to explore the potential combinatorial space of the objects present in the assembly pool toward creating novel unique objects. At this timescale, assuming there is no selectivity (emerging from the *internal* structure of the objects and their interactions) within the physical process to build a specific object or there is no *optimized process* to create a specific object, in that case, the chances of formation of all possible objects utilizing the assembly pool are equally likely. Or, even in the presence of selectivity to a specific set of objects over the others in the assembly pool, the probability of the selection of objects for the assembly process is higher, however, the assembly process is still not optimized for building a specific object. This signifies that the amount of work done to assemble a specific object (with or without selectivity) at the discovery time scale could be assumed independent of the object type if the assembly process is not optimized for creating that specific object. Considering the amount of work done by an unoptimized assembly process to create each copy of the observed object is equal, the dependence of the copy number to the quantification of Assembly should be linear ($A \propto n_i$).

The production timescale ($\tau_\mathrm{p}$) is usually specific to a given object and generates copies at the production rate which could be assumed faster than the discovery rate in the case of large copy numbers of specific objects. In this case, the production process for the specific object has been *optimized* (no competition) or at least *partially optimized* (partial competition) such that a large number of copies of an observed object can be produced. In principle, the production process of observing two copies after the discovery of an object is more significant than observing 1001 copies given that 1000 copies already exist. This is because, after the discovery of an object (its assembly pathway), it takes time to optimize the production process. At a lower copy number, when the

production process has not been optimized, the probabilities of the production of a specific object or the discovery of a new unique object could be considered similar. However, the presence of a large number of copies of a specific object represents an optimized process. Hence, for an already optimized process, the dependence of the copy number of a specific object on the overall Assembly should be logarithmic ($A \propto \log(n_i)$). Additionally, the unique objects discovered along the assembly paths can have different times scales of production and these timescales can potentially change with time due to an increase in the competition with the arrival of newly discovered objects. This suggests that the discovery time scale with an unoptimized assembly process is more suitable for quantifying the total contingency within the ensemble, hence we chose a linear dependence of the copy number of an observed object on the Assembly of the ensemble.

### 3.3 Assembly in Joint Assembly Space

For an isolated assembly path, with an exponential dependence on the assembly index and linear dependence on the copy number, for $N$ observed unique objects, the Assembly ($A$) is given by

$$A = \sum_{i}^{N} e^{a_i} \left(\frac{n_i - 1}{N_T}\right) \tag{1}$$

where $a_i$ and $n_i$ are the assembly index and the copy number of the $i^{\text{th}}$ object and $N_T$ is the total number of objects in the ensemble. The exponential $e^{a_i}$ quantifies the contingency along the path by summing the contribution at each step as +1. Along the assembly path, the information required at an assembly step to construct an object gets "stored" within an object during the assembly process, and hence can be efficiently utilized again for further construction. This suggests that at each assembly step, the assembly process utilizes the "stored" information together with the additional information to construct the object with higher assembly.

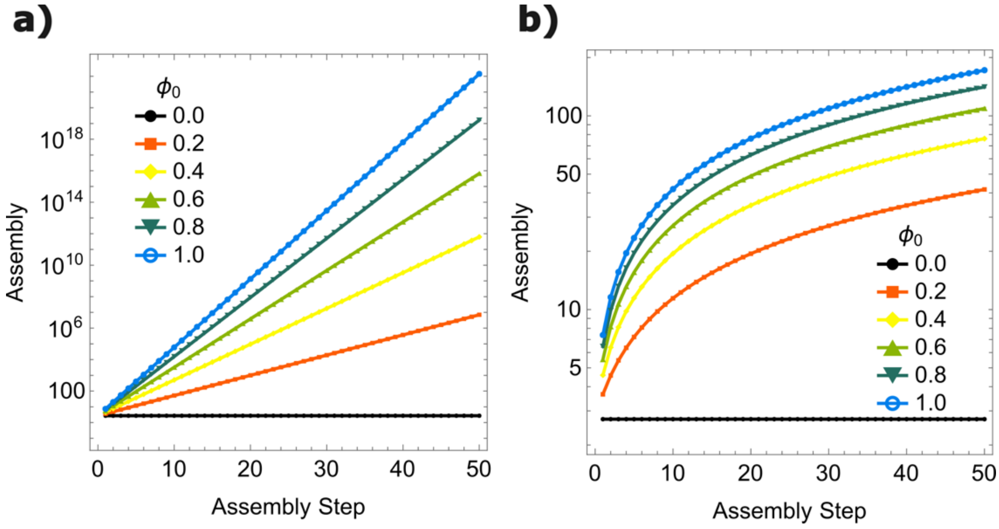


**Fig S8. Assembly of an ensemble.** (a) and (b) show the Assembly of the ensemble assuming isolated chains up to 50 assembly steps when $\phi_a$ is constant with the assembly index and when $\phi_a$ scales

with assembly index $a$ as $\phi_a = \phi_0 f^{a-1}$, where $\phi_0$ is a fraction at the first step (see legend) and $f = 0.33$ represents the increase in constraints with assembly steps. In both cases, the summation was performed up to additional 100 assembly indices, such that $a_{max} = 101$, with an initial number of objects at assembly index $a = 1$ set as $10^{12}$. Due to large initial number of objects, for simplicity all copy numbers $> 1$ including non-integer values, were considered in calculating Assembly $A$. Cases with copy numbers < 1 were excluded. With the increase in $\phi$ conversion fraction, objects at higher assembly emerge with higher copy numbers which for an isolated chain represents the efficiency of the forward process to construct complex objects.

As an example for estimating Assembly, consider a simple forward process, where at each step a fraction of objects ($\phi_a$) at assembly index $a$ combines with building blocks and transforms into a higher assembly object $a \rightarrow a + 1$ such that at assembly step $t$, the copy numbers $n_a(t)$ of objects with assembly index $a$ is given by $(1 - \phi_a)n_a(t) + \phi_{a-1}n_{a-1}(t)$ in the presence and the absence of constraints. The estimated assembly ($A$) up to 50 assembly steps in two different cases is shown in Fig. S8a and S8b.

The formulation of Assembly $A$ is general and could be modified in different ways for specific problems. Here, as one of the possible extensions, we expand the formulation to introduce strict quantification of shared paths in a joint assembly space. We define the path-dependent contribution of step $a \rightarrow a + 1$ for an object as $\prod_{j=1}^{a} p_j$, where $p_j$ is the contribution at the $j^{\text{th}}$ step. Hence, for an object with an assembly index $a$, the exponential contribution is defined as the summation of all the construction steps along the assembly path $\sum_{j=1}^{a} \prod_{k=1}^{j} p_k$. Hence, for $N$ unique objects, the generalized formulation for the assembly can be defined by modifying equation 1 as,

$$A = \sum_{i}^{N} e^{\sum_{j=1}^{a} \prod_{k=1}^{j} p_k} \left( \frac{n_i - 1}{N_T} \right) \tag{2}$$

when the chain is isolated, for each construction step $\prod_{k=1}^{j} p_k = 1$ and hence $\sum_{j=1}^{a} 1 = a$ and equation 2 simplifies to equation 1. When the chain is isolated, the *selection* process at each step along the assembly path is implicit and quantified within the assembly process. However, in the case of joint assembly space, when multiple objects coexist and have been observed, the *selection* process has explicit dependence. In the case of joint assembly space, $\prod_{j=1}^{a_i} p_j$ must quantify both the explicit *selection* and assembly process at that step, see Fig. S9(a) for comparison. To quantify the explicit selection, at a given contingent node of the assembly pathway, we assume that the contribution to the selection process scales with the number of observed paths. This is based on the fact that the higher the number of observed paths at a given contingent node, the higher the utilization and hence, the higher the selectivity has been acquired at that node. Another consideration in quantifying *selection* is the assembly step number at which selection occurs along the assembly path. With the increase in

the number of construction steps along the assembly path, the probability of the emergence of selectivity is higher due to the increase in the complexity of the structure, which is a natural process. Hence, even if the assembly process at the lower assembly index is easier, the emergence of selectivity at lower assembly indices is a unique process and must be quantified as a higher contribution as compared to selectivity at higher indices. We defined the selection contribution for the step $k-1 \rightarrow k$ as $s_k = \frac{\gamma}{k}\left(1 - \frac{1}{p_{k-1\rightarrow k}}\right)$, where $p_{k-1\rightarrow k}$ is the total number of the observed paths along the assembly path at the $k^{\text{th}}$ contingent node and $\gamma$ is a constant, which quantifies the relative contribution of *explicit selection* over the construction as a given step. So, with the total contribution (construction (+1) and selection ($s_k$)) from the $k^{\text{th}}$ node is $1 + \frac{\gamma}{k}\left(1 - \frac{1}{p_{k-1\rightarrow k}}\right)$ and the path-dependent contribution of step $a \rightarrow a+1$ is quantified by $\prod_{k=1}^{a}\left(1 + \frac{\gamma}{k}\left(1 - \frac{1}{p_{k-1\rightarrow k}}\right)\right)$. So, for an object with an assembly index $a$, in the case of joint assembly space, the exponential contribution to the can be modified as $\sum_{j=1}^{a}\prod_{k=1}^{j}\left(1 + \frac{\gamma}{k}\left(1 - \frac{1}{p_{k-1\rightarrow k}}\right)\right)$. With an addition of $a_{ch}$ as the characteristic assembly constant (refers to the assembly index of building blocks or fundamental particles), the generalized formulation of the Assembly equation for $N$ unique objects in the joint assembly space is given by

$$A(t) = \sum_{i=1}^{N} e^{\left(\sum_{j=1}^{a_i}\prod_{k=1}^{j}\left(1+\frac{\gamma}{k}\left(1-\frac{1}{p_{k-1\rightarrow k}}\right)\right)-a_{ch}\right)}\left(\frac{n_i(t)-1}{N_T}\right) \tag{3}$$

As an example, consider three cases as shown in Fig. S9(b) with one isolated and two shared assembly processes. In the isolated assembly path, with $a_{ch} = 0$, the total contribution in the exponential for the object $a_5$ is 5. However, in case 2 with one shared event at $a_2$, the total contribution in the exponential for the object $a_{521}$ is stepwise along the path is $\left(1 + (1\times 1) + \left(1\times 1\times\left(1+\gamma\frac{1}{6}\right)\right) + \left(1\times 1\times\left(1+\gamma\frac{1}{6}\right)\times 1\right) + \left(1\times 1\times\left(1+\gamma\frac{1}{6}\right)\times 1\times\left(1+\gamma\frac{1}{10}\right)\right)\right)$. Similarly, in case 3, the total contribution in the exponential for the object $a_{521}$ is $\left(1 + (1\times 1) + \left(1\times 1\times\left(1+\gamma\frac{2}{9}\right)\right) + \left(1\times 1\times\left(1+\gamma\frac{2}{9}\right)\times 1\right) + \left(1\times 1\times\left(1+\gamma\frac{2}{9}\right)\times 1\times\left(1+\gamma\frac{1}{10}\right)\right)\right)$. It should be noted that the above formulation is one of the potential extensions in quantifying Assembly over an ensemble to quantify the effect to shared assembly space.

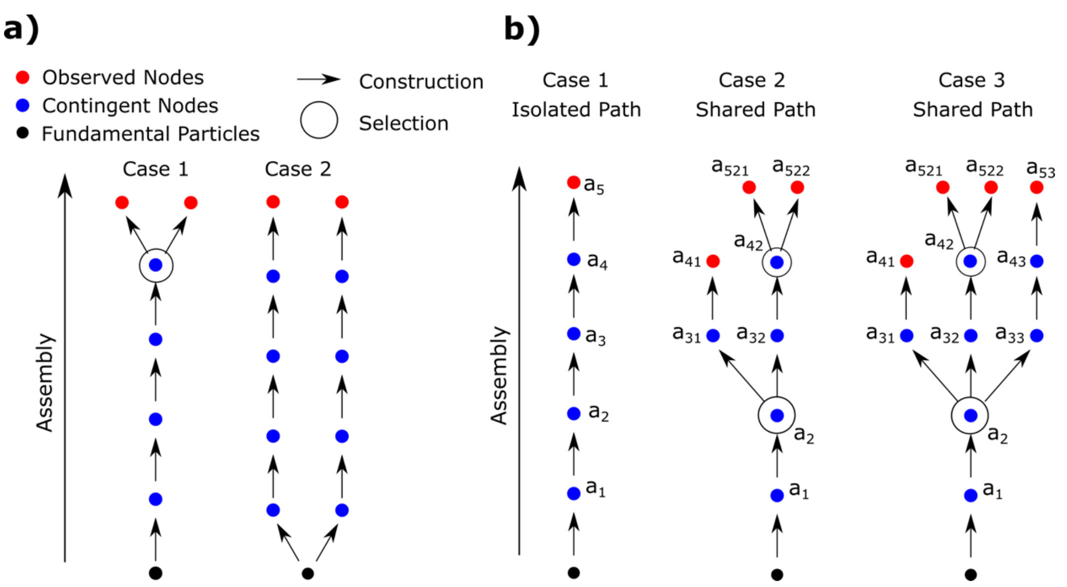


**Fig. S9 Quantifying the Assembly of an ensemble with objects in joint assembly space** (a) Joint Assembly Space of two observed objects in two different cases, left: splitting at later assembly steps right: splitting at the earlier assembly steps, (b) Three different cases with different selection and construction processes. Case 1: Isolated chain with no explicit selection, Case 2 & 3: Shared assembly paths with explicit selection.

## 4. Assembly Universe and super-exponential expansion

### 4.1 Scaling in assembly universe

We analyze the expansion of the number of all possibilities with the assembly index, which we call the Assembly Universe. A simple model of expansion, utilizing the algorithmic nature of assembly processes, assumes that the number of objects $N_a$ with a given Assembly Index $a$ can combine with themselves and with lower Assembly Index objects ${N_a}^{1+\delta}$ ways to make objects with assembly index $a+1$, leading to the recurrence relation $N_{a+1} \sim {N_a}^{1+\delta}$ , which, in turn, describes a double-exponentially expanding Assembly Universe,

$$N_a \sim {N_0}^{[(1+\delta)^a]} = e^{\nu e^{\mu a}} \quad (4)$$

With $\nu = ln\, N_0$ and $\mu = ln\,(1+\delta)$. $\delta$ can be interpreted as the scaling of the average number of other objects $\langle k \rangle_a \sim {N_a}^{\delta}$ a particular object with assembly index $a$ can combine with to form an object with assembly index $a+1$, recovering the recurrence relation above, $N_{a+1} \sim N_a \langle k \rangle_a$. The double-exponential expansion, arising from a constant (i.e., assembly index-independent) expansion rate $\delta$>0, is shown in Fig S10a, consistent with statistics of novelty generation in thermodynamics of structure forming systems that do not include physical constraints emerging from the past history of

what has been assembled and the intrinsic assembly of the objects that exist. The expansion of the Assembly Universe is very fast, even when the expansion rate $\delta$ is small. We further analyze a generalized growth model where the expansion rate $\delta(a;\eta,\lambda)$ decreases with complexity as $\delta = \frac{\eta}{a^{\lambda}}$. This is a phenomenologically motivated model of the increasing number of constraints imposed by more complex objects being present in the system. In this generalized model, the Assembly Universe grows as $N_a \sim N_0^{\prod_{k=1}^{a-1}\frac{1}{k^{\lambda}}(\eta+k^{\lambda})}$, which simplifies to $N_a \sim N_0^{\frac{\Gamma(a+\eta)}{\Gamma(a)\,\Gamma(1+\eta)}}$ when $\lambda = 1$, i.e., for expansion rate $\delta = \eta a^{-1}$. This growth class is illustrated in Fig. S10b. When $\lambda > 1$, that is, when the expansion rate decays faster than inversely proportional to assembly index, the Assembly Universe saturates at $\lim_{a\to\infty} N_a = N_0^{\frac{\sinh[\pi\sqrt{\eta}]}{\pi\sqrt{\eta}}}$, as Fig. S10c demonstrates. The generalized model suggests that while the Assembly Universe grows very fast without emerging constraints, when constraints increase with complexity this growth can be tamed effectively, leading to sub-exponential growth or even a saturating Assembly Universe.

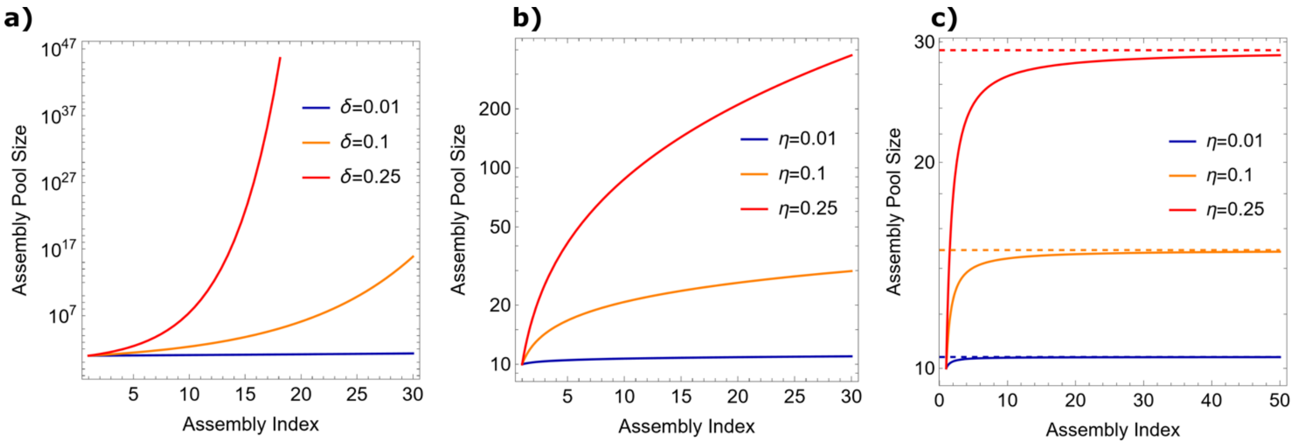


**Fig. S10 Expansion of Assembly Universe.** (a) Shows the assembly pool size versus the maximum assembly index in the absence of constraints, which is defined by a double exponential relation at different expansion factors ($\delta$). (b) & (c) Shows the assembly pool size versus the maximum assembly index with emerging constraints $\delta \sim \frac{\eta}{a^{\lambda}}$ at $\lambda = 1$ & $\lambda = 2$. The dotted lines are the limiting values when $a \to \infty$. In all cases, the total number of initial objects was $N_0$ was assumed as 10.

Additionally, an approximate argument that supports a super-exponential growth in generic systems that are *both combinatorial and compositional* is the following. Objects of size $S$ come in an exponential multiplicity (since objects are combinatorial), $N(S) \sim e^{\sigma S}$. The minimum number of steps $a$ needed to construct an object with size $S$ is somewhere between $a(S) \sim \log S$ and $a(S) \sim S$, corresponding to constructing by recursive doubling and constructing element-by-element, respectively. Inverting $a(S)$ gives an $S(a)$ that is between linear and exponential. Taken together, $N(S(a)) \sim e^{\sigma S(a)}$, which scales between exponential $N_a \sim e^{\sigma a}$ and double exponential, $N_a \sim e^{\sigma e^{\tau a}}$. A better approximation can be given by taking into account the *distribution* of assembly indices of

objects with size S, $P(a|S)$, which can be inverted following Bayes' rule as $P(S|a) = P(a|S)P(S)/Z$, where $P(S) \sim N(S) \sim e^{\sigma S}$ as above, and $Z$ ensures normalization. $P(S|a)$ then can be used to calculate the scaling of Assembly Universe as $N_a \sim \sum_S P(S|a)\ e^{\sigma S}$.

## 5. Assembly Possible and Assembly Contingent

### 5.1 Dynamics of Assembly Possible

Quantifying selection and design in spaces of hierarchically modular structures are one of the main objectives of this study. It is, therefore, crucial to understand what exploration dynamics to expect in the *absence* of selection and design, which we call *undirected* dynamics. What is explored via undirected dynamics in the assembly spaces is the possibilities that can be realized taking into account the contingency of the dynamics but nothing else. That is, we account for the past history of what has been assembled to constrain the future space, but do not include biases that arise in selection. This dynamically expanding subset of the Assembly Universe is what we call Assembly Possible.

Microscopically, the Assembly Possible is determined by both the structure of the Assembly Universe (all assembly operations and what they make) and the way "undirectedness" is mathematically formulated. For example, undirected could correspond to the selection of two objects from a pool and combining them in one uniformly selected way or selecting uniformly among the triplets (*two objects* to combine, and a *way* to combine them). The latter more heavily weights objects that can be combined in more possible ways as compared to the former. Instead of going into the details of specific systems, here we formulate a simple phenomenological model to define the class of undirected assembly in a generic (but approximate) way as follows. When an object with Assembly Index $a$ combines with *its own contingent history*, its Assembly Index increases by one, $a \rightarrow a + 1$. There are two exceptions having opposing effects. If the resulting object can be made via another, shorter path(s), its Assembly Index will be smaller than $a + 1$. If the object combines with another which is not in its own contingent history, the combination of them might have an Assembly Index that is larger than $a + 1$ (except if there is a shorter path as discussed above). These two effects may or may not statistically cancel. In systems that we study here (integers, polymers, graphs, molecules), they approximately do, as we show below. Another assumption behind the dynamical model of undirected dynamics is that it is microscopically driven by a stochastic rule that uses existing objects uniformly: the probability of choosing an object with Assembly Index $a$ to be combined with another is proportional to $N_a$, the number of objects with Assembly Index $a$. These two arguments suggest the following dynamics generating the Assembly Possible:

$$\frac{dN_{a+1}(t)}{dt} = k_d\ N_a(t) \tag{5}$$

which can be solved analytically, and the solution is given by,

$$N_a(t) = N_0 \frac{(t\, k_d)^a}{\Gamma(1+a)} = N_0 \frac{(t\, k_d)^a}{a!} \tag{6}$$

where, $k_d$ is the discovery or expansion rate and $N_0$ is the initial number of unique objects. The derivative $\frac{dN_a(t)}{da}$ is given by,

$$\frac{dN_a(t)}{da} = N_0 \frac{(t\, k_d)^a}{\Gamma(1+a)} (\gamma - H_a + \log(t\, k_d)) \tag{7}$$

where, $\gamma$ is the Euler's constant, and $H_a$ is the $a^{\text{th}}$ Harmonic Number. Using the asymptotic expansion, the Harmonic Number $H_a$ with an asymptotic expansion, $H_a \sim \log(a) + \gamma + \frac{1}{2a} - \sum_{k=1}^{\infty} \frac{B_{2k}}{2k a^{2k}}$ where, $B_k$ are the Bernoulli numbers. Approximating the Harmonic number as $H_a \sim \log(a) + \gamma$, the time-dependent assembly index at which the maximum assembly index unique object occurs is given by $a_{max} = t\, k_d$.

The number of unique objects at the assembly value $a_{max}$ is $N_a(t) = \frac{N_0 (k_d\, t)^{k_d\, t}}{\Gamma(1+k_d\, t)}$. Adding an extra correction factor with $H_a \sim +\gamma + \frac{1}{2a}$ and excluding the infinite series, the assembly at maximum unique objects is given by $a_{max} = e^{\text{ProductLog}\left(-\frac{1}{2k_d\, t}\right)} k_d\, t$ where ProductLog is the principle solution for $w$ in $z = we^{\text{w}}$. The number of unique objects at the assembly value $a_{max}$ is $N_a(t) = N_0 \frac{(k_d t)^{e^{\text{ProductLog}\left(-\frac{1}{2k_d\, t}\right)} k_d\, t}}{\Gamma(1+e^{\text{ProductLog}\left(-\frac{1}{2k_d\, t}\right)} k_d\, t)}$.

As a proof for the exponential expansion of assembly possible, applying Sterling's approximation on equation 6 gives, $N_a(t) = N_0 \frac{(t\, k_d)^a}{a!}$ , $ln\left(\frac{N_a(t)}{N_0}\right) = a\, ln(k_d t) - a\, ln\, a + a = a\,(ln\,(k_d t) - ln\, a + 1) \approx a\, ln\, \bar{a}$. Hence, $N_a \approx N_0\, exp(a\, ln\, \bar{a})$. This shows that the dynamics of Assembly Possible expand exponentially. The dynamics of Assembly Possible is shown in Figures S11a and S11b.

### 5.2 Dynamics of Assembly Contingent

By introducing the selection parameter $\alpha$ with $0 \leq \alpha \leq 1$, the equation of assembly contingent is given by,

$$\frac{dN_{a+1}(t)}{dt} = k_d(N_a(t))^{\alpha} \tag{8}$$

The selection parameter is a phenomenological and observable parameter that characterizes the directedness or selectivity of the physical processes. Higher values of $\alpha \approx 1$ describe the near-random dynamics similar to the Assembly Possible, whereas lower $\alpha$ values, $\alpha \approx 0$, describe selective and goal-directed processes. The equation can be solved analytically for different assembly indices using Wolfram Mathematica 13.0, and the solutions at different assembly indexes are given by,

$$N_1(t) = (N_0)^{\alpha} k_d\, t \tag{9}$$

$$N_2(t) = (N_0)^{\alpha^2} \frac{(k_d\, t)^{1+\alpha}}{1+\alpha} \tag{10}$$

$$N_3(t) = (N_0)^{\alpha^3} (1+\alpha)^{-\alpha} \frac{(k_d\, t)^{1+\alpha+\alpha^2}}{1+\alpha+\alpha^2} \tag{11}$$

$$N_4(t) = (N_0)^{\alpha^4} \frac{(1+\alpha)^{-1-\alpha^2}(1+\alpha+\alpha^2)^{-\alpha}(k_d\, t)^{(1+\alpha)(1+\alpha^2)}}{1+\alpha^2} \tag{12}$$

$$N_5(t) = (N_0)^{\alpha^5} \frac{t\,(1+\alpha)^{-\alpha(1+\alpha^2)}(1+\alpha^2)^{-\alpha}(1+\alpha+\alpha^2)^{-\alpha^2} k_d (k_d\, t)^{\alpha(1+\alpha)(1+\alpha^2)}}{1+\alpha+\alpha^2+\alpha^3+\alpha^4} \tag{13}$$

Based on the solutions $N_1(t) - N_5(t)$ given by equations $9-13$, the generalized solution of the equation can be derived. With $N_0$ unique objects at $t = 0$, the number of unique objects of assembly index $a$ at time $t$ in the assembly contingent is given by,

$$N_a(t) = \frac{(N_0)^{\alpha^a}}{\sum_{k=0}^{a-1} \alpha^k} \left( \prod_{j=1}^{a-1} \left( \sum_{k=0}^{a-1-j} \alpha^k \right)^{-\alpha^j} \right) (k_d\, t)^{\sum_{k=0}^{a-1} \alpha^k} \tag{14}$$

which, could be simplified further to

$$N_a(t) = (N_0)^{\alpha^a} \frac{\alpha - 1}{\alpha^a - 1} \left( \prod_{j=1}^{a-1} \left( \frac{\alpha^{a-j} - 1}{\alpha - 1} \right)^{-\alpha^j} \right) (k_d\, t)^{\frac{\alpha^a - 1}{\alpha - 1}} \tag{15}$$

Equation 15 simplifies to equation 6 when $\alpha = 1$, which is equivalent to the dynamics of Assembly Possible. Fig. S11c shows the distribution of the number of unique objects *vs.* assembly index at different selection parameters at a given time t ($t = 10$, dimensionless units). The peak of the distribution falls and the distribution of unique objects at higher assembly increases with the increase in $\alpha$ demonstrating the transition from Assembly Possible to Assembly Contingent. The total number of objects with time up to 100 dimensionless time steps is shown in Fig. S11d.

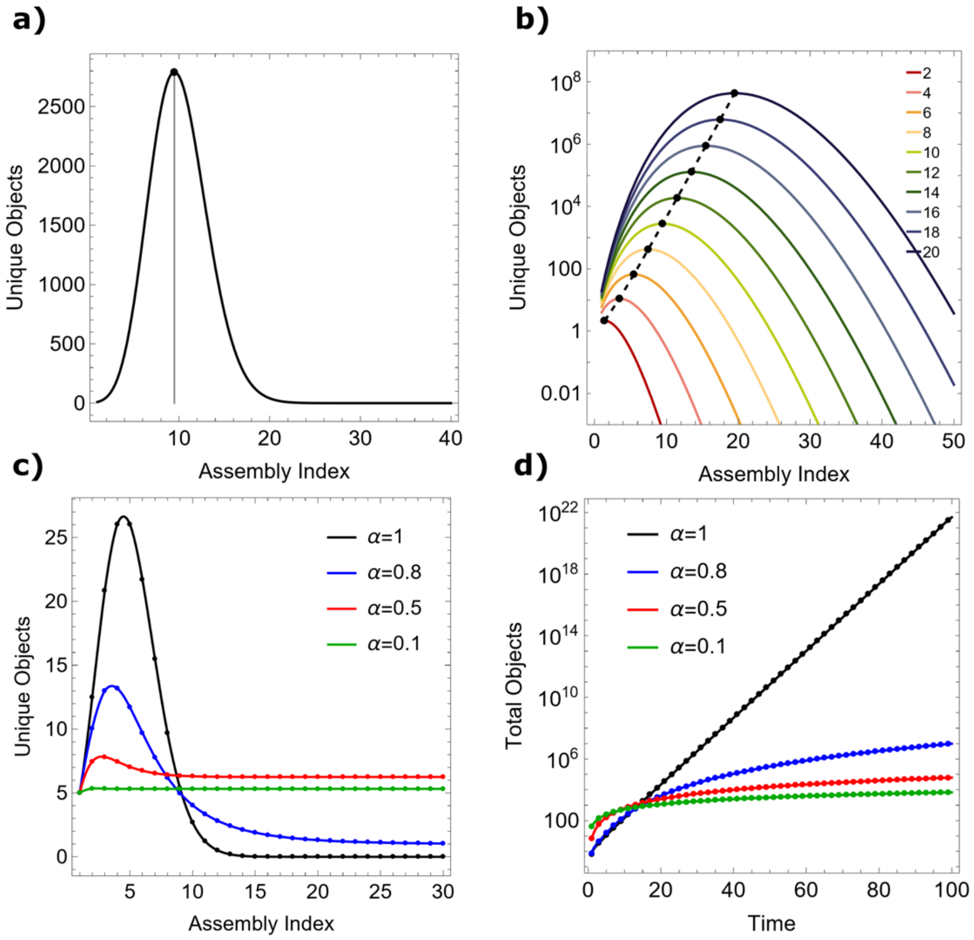


**Fig. S11 Dynamics of Assembly Possible and Assembly Contingent.** (a) The distribution of the number of unique objects *vs.* assembly index with $N_0 = 1, \kappa = 1, t = 10$ describing Assembly Possible. (b) The distribution of the number of unique objects with the same parameters at different dimensionless times in the range $[2, 20]$ with a typical assembly index (maximum of the distribution) is shown by the dashed line. (c) The distribution of the number of unique objects versus the assembly index with $N_0 = 1, \kappa = 0.5, t = 10$ with different selection parameters $\alpha$ describing assembly contingent. (d) The total number of unique objects counted to assembly index 100 *vs.* dimensionless time with the same parameters as in (c).

## 6. Joint Assembly Space in a Linear Chain Model

### 6.1 Joint Assembly Space of observed objects

The Joint Assembly Space defines the combined assembly space formed when multiple objects coexist together. In principle, it represents the shortest pathway to construct multiple coexisting objects. Given $n$ independently observed objects with their assembly paths defined by

$G_1, G_2, G_3 \dots G_n$. Here, for simplicity, we approximate the joint assembly space $J$ for the observed objects, defined as $J = G_1 \cup G_2 \cup G_3 \dots \cup G_n$. As an example, consider two linear chains with chain lengths 14 and 31. The independent assembly paths ($G_1$and $G_2$) for these linear chains can be estimated from the method described in the previous section (Section 2) with assembly indices 4 and 5. If the two chains coexist, the joint assembly space can be approximated as $J = G_1 \cup G_2$. The part of the assembly path within the joint assembly space shared by the observed objects is called shared assembly space. As an example, for two linear chains of lengths 14 and 31, the isolated assembly paths $G_1$ and $G_2$ for the two chains are shown in Fig. S12 (a & b). The combined joint assembly space when the two chains coexist is shown in Fig. S12c. The nodes {1, 2, 3, 4, 7} are shared between the two observed chains and comprise the shared assembly space (shown in green).

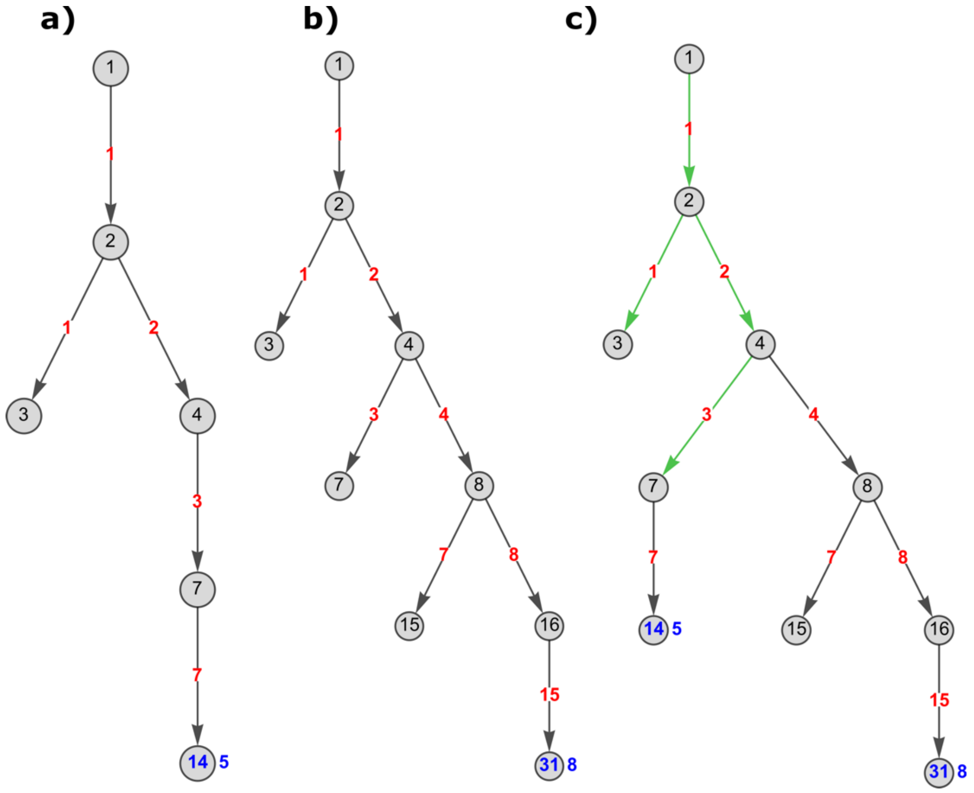


**Fig. S12 Joint Assembly Space of two observed linear chains.** (a) and (b) shows the isolated assembly paths of observed linear chains of lengths 14 and 31. (c) shows the joint assembly space when chains 14 and 31 coexist. The nodes number represents the length of the polymeric chains. The nodes in blue represents observed nodes (with assembly index on the right), in black represent contingent nodes (not observed but part of assembly space), and in red represent combined nodes (joined together). The shared assembly space is shown in green.

### 6.2 Joint Assembly Space in the forward process

Similar to in SI Section 1, here we describe a forward process which combines objects in the assembly pool to create objects of higher assembly. Here, to distinguish the undirected process from the directed process, we consider forward processes in two different ways,

1. **Undirected Exploration** is when two objects from the assembly pool are chosen randomly and combined to create a new object. The newly generated object is then added to the list of the assembled objects and if it is unique and previously does not exist in the assembly pool, it is also added to the assembly pool.

2. **Directed Exploration** where the last object (longest chain) is selected from the assembly pool and combined with the randomly chosen object to create the new object. Similar to the undirected exploration, the newly generated object is then added to the list of assembled objects and if it is unique and previously does not exist in the assembly pool, then it is also added to the assembly pool.

After iterating the undirected and directed exploration over a given number of steps, the assembly paths of all the emerged structures in the assembly pool are generated. From the list of assembly paths, the joint assembly space is generated by taking the pairwise union over all the assembly paths. $J = \left(\left((G_1 \cup G_2) \cup G_3\right) \ldots \cup G_N\right)$, where $G_n$ represents the assembly path of the $n^{\text{th}}$ object with total $N$ objects in the assembly pool. The undirected and directed exploration of the assembly space of linear chains was implemented in Mathematica 13 and simulated up to $10^4$ steps. The exploration space expands rapidly to higher assembly indices, especially in the case of directed process. Fig. S13 shows the joint assembly space of all the linear chains generated in undirected and directed processes simulated up to 20 assembly steps.

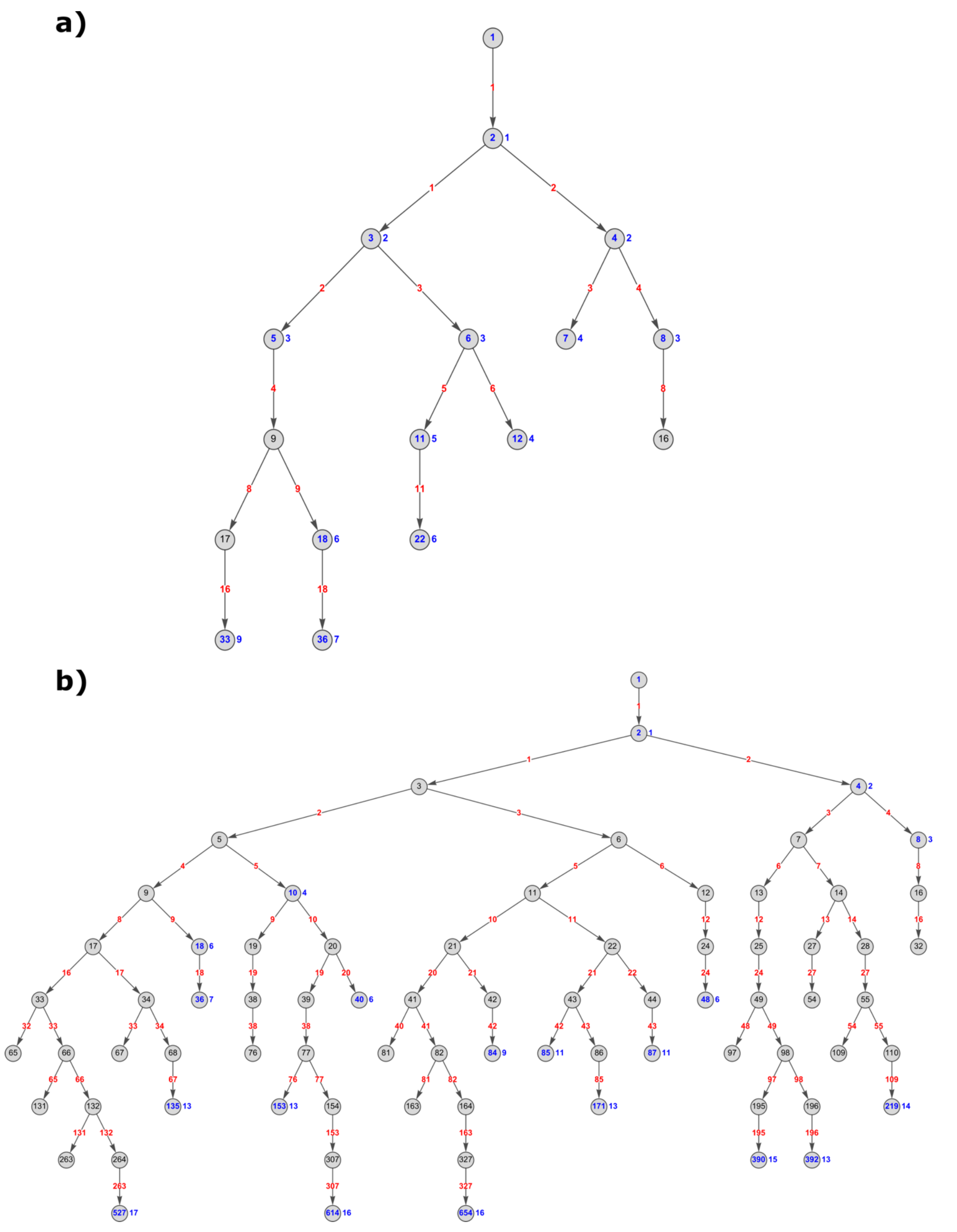


**Fig. S13 Joint Assembly Space for linear chains.** (a & b) Joint assembly space of a forward process with undirected exploration and directed exploration after 20 assembly steps. The node number represents the length of the polymeric chains. The nodes in blue represent observed nodes (with assembly index on the right), in black represent contingent nodes (not observed but part of assembly space), and in red represent combined nodes (joined together).

## 7. Distribution of unique objects in a forward process with different selectivity

We extended the linear polymeric chain model by allowing directedness to emerge from a physical process more naturally. In this model, at each step, the linear chains in the assembly pool are sorted based on their lengths, and then a subset is selected for further exploration using forward dynamics. At each assembly step, after sorting, a subset is selected based on the selection index ($\delta$) which represents the fraction of the assembly pool from which the polymeric sequences will be combined. From the selected subset, two polymeric chains are randomly selected and combined. The selection index represents the selective process that determines the fraction of the assembly pool with sorted chain lengths that can be used by a forward process for the growth process. This selective process resembles a physical process which *separates* the objects (polymeric sequences) based on the length of linear chains and *selects* among them for the assembly process. The exploration in assembly space (see Fig. S14) in the absence of selection ($\delta = 1$) is shown below and was implemented in Wolfram Mathematica 13. The higher the selection index the lower the selectivity as it represents the fraction of the sorted linear chains which can be used for the further assembly process.

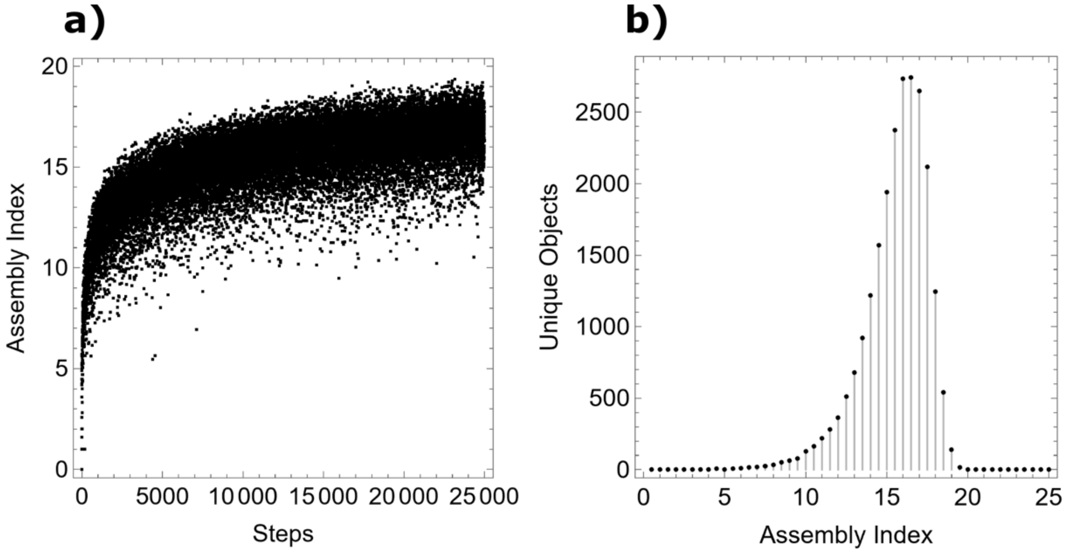


**Fig. S14 Exploration in assembly space.** (a) Example exploration of assembly space of linear chains with undirected dynamics (with selection index $\delta = 1$). The figure shows the assembly index (approximated by $\log_2(n)$) with assembly steps up to 25000 steps. (b) Distribution of unique objects *vs.* assembly index within the assembly pool.

As shown previously, for a linear polymeric chain comprise of $n$ monomeric units, the assembly index ($a_n$) can be approximated as $\log_2(n)$ in the leading order. Fig. S15a. Fig. S15b shows the growth of assembly index in the assembly space at various selection indices ($\delta$) in the range ($10^{-4}$ – 0.8) over $5\times10^4$ assembly steps. Each point represents an addition step in the formation of polymeric chains. A smaller selection index signifies larger information processing in a physical system (by

sorting and selecting a smaller subset) leading to stronger selectivity within the assembly pool. With the increase in the selectivity in the system (decrease in the value of selection index) the peak value of the assembly index distribution of unique linear chains falls however, the number of unique linear chains observed at higher assembly index increases, see Fig. S15c and S15d. This shows that the observed distribution of the unique objects over the range of assembly indicates the complexity of the system, and the amount of contingency utilized to create the observed distribution is signified by the selection index.

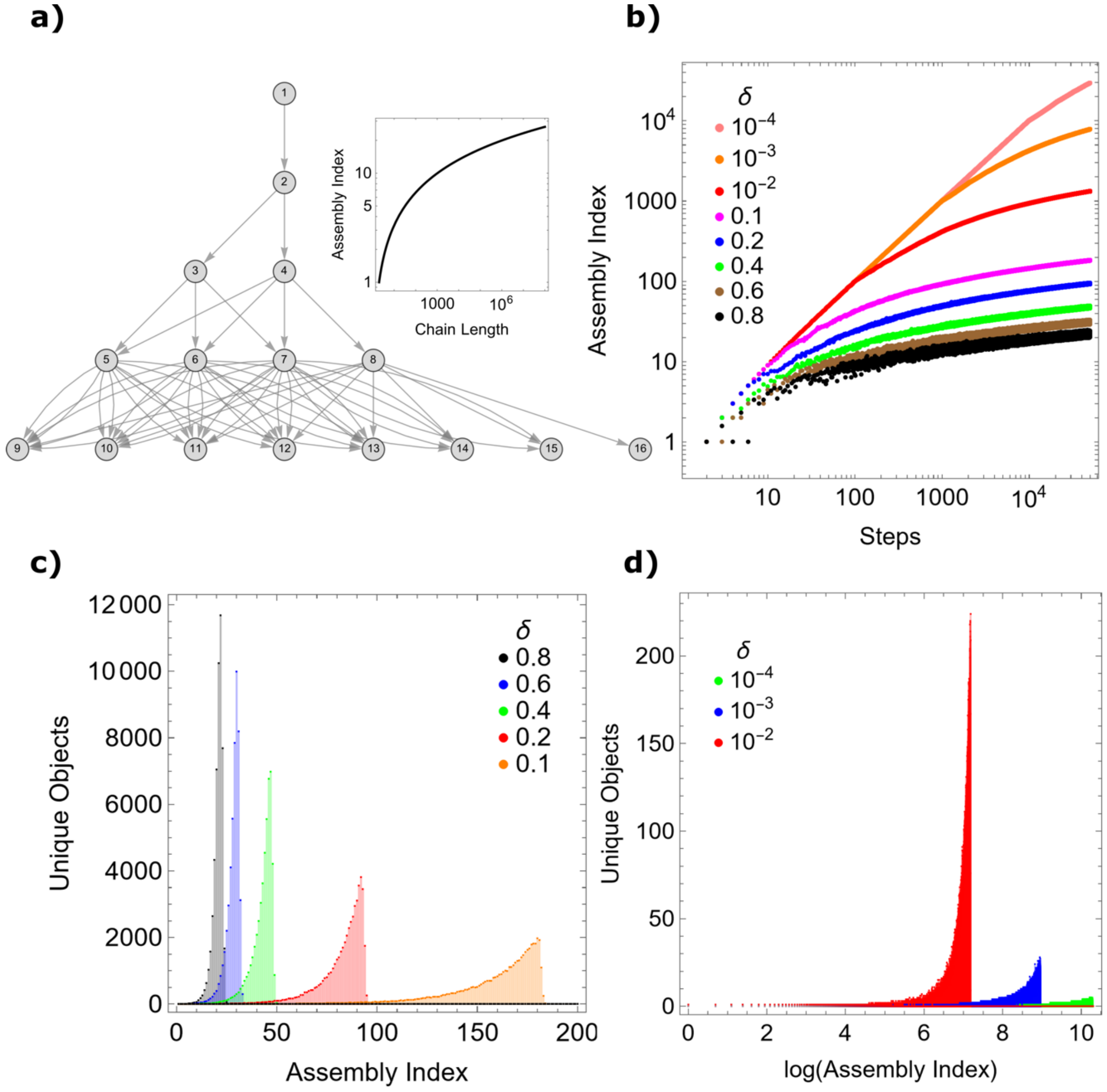


**Fig. S15 Linear Chain Model as a forward process at different selection indices** (a) Forward process describing the assembly paths of possible linear chains. The inset plot shows the assembly index *vs*. length of the linear chains, (b) the growth of assembly index (approximated as $\log_2(n)$) over $5\times10^4$ steps at different selection indices ($\delta$), (c) and (d) shows the distribution of observed unique objects over the assembly index after $5\times10^4$ assembly steps at different assembly indices.

## 8. Assembly Contingent combined with mass transfer kinetics

Consider a forward assembly process where the copy number of the emerging species follows the homogeneous kinetics,

$$N_0(t)n_0(t) \rightarrow N_1(t)n_1(t) \rightarrow N_2(t)n_2(t) \rightarrow \cdots N_a(t)n_a(t) \rightarrow N_{a+1}(t)n_{a+1}(t) \ldots$$

where $N_a(t)$ represents the number of unique objects at the assembly index and $n_a(t)$ as the copy number of each unique object at assembly index $a$. We assume that the forward rate constants decrease with the increase in assembly index of the emerging structures by a factor $\beta$ at each step and $k_{p0}$ is the initial production rate constant ($k_p$) at the first step ($n_0(t) \rightarrow n_1(t)$), hence for assembly step $a \rightarrow a+1$, $k_a = k_{p0}\beta^{a-1}$. This decrease in the rate constant with assembly steps is introduced in the formulation, as with an increase in the assembly index, the object becomes more complex, and interactions become more specific, leading to a decrease in production rate. For simplicity, assuming all objects at the assembly $a$ have the same copy number (for the purpose of understanding discovery-production dynamics), the total number of objects at the assembly index $a$ at a given time $t$ is given by $N_a(t)n_a(t)$. This is an over simplification of a more complex network of growing branches where a single branch is represented by $n_0(t) \rightarrow n_{1,i}(t) \rightarrow n_{2,j}(t) \rightarrow n_{3,k}(t) \rightarrow \cdots n_{a,p}(t) \rightarrow n_{a+1,q}(t) \ldots$, where $n_{a,p}(t)$ is the copy number of $p^{\text{th}}$ object at assembly index $a$. Is it understandable that at the same assembly index, the objects along a single branch which have been discovered earlier should have significantly different copy number than the objects which have been discovered later. This assumption does not hold in a physical system; however, it simplifies the formulation drastically to highlight key features of discovery-production dynamics. Additionally, we like to highlight the significance of the time evolution integrated quantity $A$, instead of developing a more complicated model. The kinetic rate equation describing the time-dependent copy numbers for emerging objects is given by,

$$\frac{d}{dt}N_a(t)n_a(t) = -k_{p0}\beta^a N_a(t)n_a(t) + k_{p0}\beta^{a-1}N_{a-1}(t)n_{a-1}(t) \tag{16}$$

and,

$$N_a(t)\frac{dn_a(t)}{dt} + n_a(t)\frac{dN_a(t)}{dt} = -k_{p0}\beta^a N_a(t)n_a(t) + k_{p0}\beta^{a-1}N_{a-1}(t)n_{a-1}(t) \tag{17}$$

Using the forward dynamics equation for undirected or directed discovery process, the rate of formation of unique objects at assembly $a$ is given by,

$$\frac{dN_a(t)}{dt} = k_d(N_{a-1}(t))^{\alpha} \tag{18}$$

Substituting equation (18) in (17),

$$N_a(t)\frac{dn_a(t)}{dt} + n_a(t)k_d(N_{a-1}(t))^{\alpha} = -k_{p0}\beta^{a}N_a(t)n_a(t) + k_{p0}\beta^{a-1}N_{a-1}(t)n_{a-1}(t) \tag{19}$$

The equation can be simplified to,

$$\frac{dn_a(t)}{dt} = -\left(k_{p0}\beta^{a} + k_d\frac{(N_{a-1}(t))^{\alpha}}{N_a(t)}\right)n_a(t) + k_{p0}\beta^{a-1}\frac{N_{a-1}(t)}{N_a(t)}n_{a-1}(t) \tag{20}$$

Equation (20) is the combined governing equation which describes the dynamics of discovery and production at any assembly index with time. In principle, equation (20) can be solved together with equation (15) which is the general solution of forward dynamics equation (8). In all cases, we assume $N_0(t)$ which describes the initial number of unique objects at any assembly index at $t = 0$ as 1, but with copy number 0. In the case, when the forward process performs an undirected discovery, $\alpha = 1$, the equation (20) can be simplified by substituting $\frac{N_{a-1}(t)}{N_a(t)} = \frac{a}{t\,k_d}$,

$$\frac{dn_a(t)}{dt} = -\left(k_{p0}\beta^{a} + \frac{a}{t}\right)n_a(t) + k_{p0}\beta^{a-1}\frac{a}{k_d\,t}\,n_{a-1}(t) \tag{21}$$

The combined set of equations with the initial conditions can be solved analytically to an extent, however, with the increase in the assembly index, the solution becomes too complicated (see Mathematica Notebook at https://github.com/croningp/assemblyphysics ). As an example, we solved equations (15) and (20) together with $\alpha = 0.5$, the maximum assembly index $a_{max} = 25$, $k_d = 1 \times 10^{-4}$, $k_{p0} = 1 \times 10^{-5}$, $\beta = 0.5$, and the initial copy number of objects at $n_0(0) = 1 \times 10^{12}$ and $N_0(0) = 1$ (only one type of object). It should be noted that these initial objects (assumed assembly index $a = 0$) $n_0(0)$ are not the only building blocks consumed to increase the assembly process. These are the building blocks governs the kinetics. We assume an infinite pool of additional building blocks exists which gets consumed along the assembly pathways, and the production kinetics is independent of them. The equations were solved numerically using the NDSolve function in Wolfram Mathematica 13. The time dependence of the total copy number of all the objects $N_a(t)n_a(t)$ and copy number of each object (assuming equal copy number of all unique objects at a given time for

each assembly index) $n_a(t)$ at an assembly index $(a)$ up to the maximum assembly index $a_{max} = 25$ is shown in Fig. S16. It is important to note that as both discovery and production are continuum models which means both number of objects and their copy numbers can have values less than 1. When the number of objects is less than one, the corresponding copy numbers are not real. So, we estimated the time at which number of unique objects at each assembly index is equal to one. The maximum time over all the assembly indices was set as the minimum time for plotting all the figures representing copy numbers. The key feature we like to observe here is that the faster number of unique objects increases, faster their copy number falls (less than one), and the forward process cannot propagate to higher assembly indices, and this can be captured by calculating Assembly of the ensemble. The equal copy number is only an assumption to observe the features of a physical process represented by discovery and production dynamics; it does not represent a realistic case where strong inhomogeneity in the copy number could exist among objects at the same assembly index due to difference in the discovery time for objects. Additionally, it is important to note that this model is a simple continuum description of the physical phenomena involving discovery and mass transfer kinetics which could lead to copy number values of less than one. This is the property of the continuum model and in principle, a model with stochastic chemical kinetics can be applied to generate a more realistic case. However, the continuum model provides important insights into the discovery and production kinetics,

1. The model can predict the time-dependent discovery of unique objects at a given assembly index and its growth dynamics in a forward process where *discovery* and *production* occur simultaneously.

2. As the complexity of the object is proportional to its assembly index, the model shows the decrease in the maximum possible copy number with the increase in assembly index due to a decrease in the production rate kinetics with the increase in assembly step.

3. The observed copy number of a given object at an assembly index strongly depends on the number of existing unique objects at a given time which is governed by the discovery dynamics (discovery rate constant $k_d$ and the selection parameter $\alpha$).

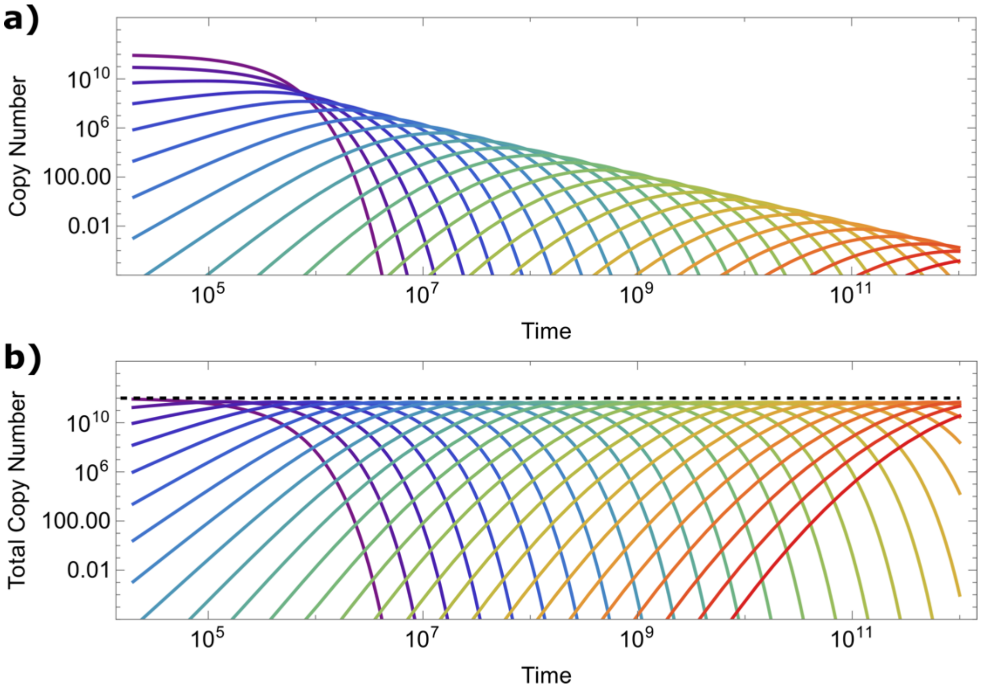


**Fig. S16 Time dependence of copy number in a forward process.** (a) Copy number of individual objects (assumed as equal in the model) at different assembly indices in the range 0–25 (dark purple to orange). (b) Total copy number of all the objects at different assembly indices in the range 0–25. The horizontal dashed black line is the total number of all the objects observed at a given time which is equal to the initial number of fundamental particles $n_0(0) = 1 \times 10^{12}$ taking part in production kinetics demonstrating that the total number of objects are conserved in the formulation.

From the time-dependent data of the number of unique objects and their copy number in a forward process, we could also define a *threshold time* which is the time at which the copy number of objects reaches the threshold value to be detected or measured. As an example, we solve equations 15 and 20 numerically with various $\alpha$ values $\{0.001, 0.2, 0.5, 0.8, 1.0\}$ with parameters $a_{max} = 25$, $k_d = 1 \times 10^{-4}$, $k_p = 1 \times 10^{-5}$, $\beta = 0.5$, $n_0(0) = 1 \times 10^{12}$, $N_0(0) = 1$ with a total dimensionless time $1 \times 10^{9}$. As a detection limit set by a measurement, we assume the required threshold copy number to be detected is 10. In each case, wherever possible the threshold time was estimated by finding intercept between the time dependent copy number for objects at assembly indices and the threshold value. Fig. S17 shows the copy number of each unique object together with the threshold time for detection.

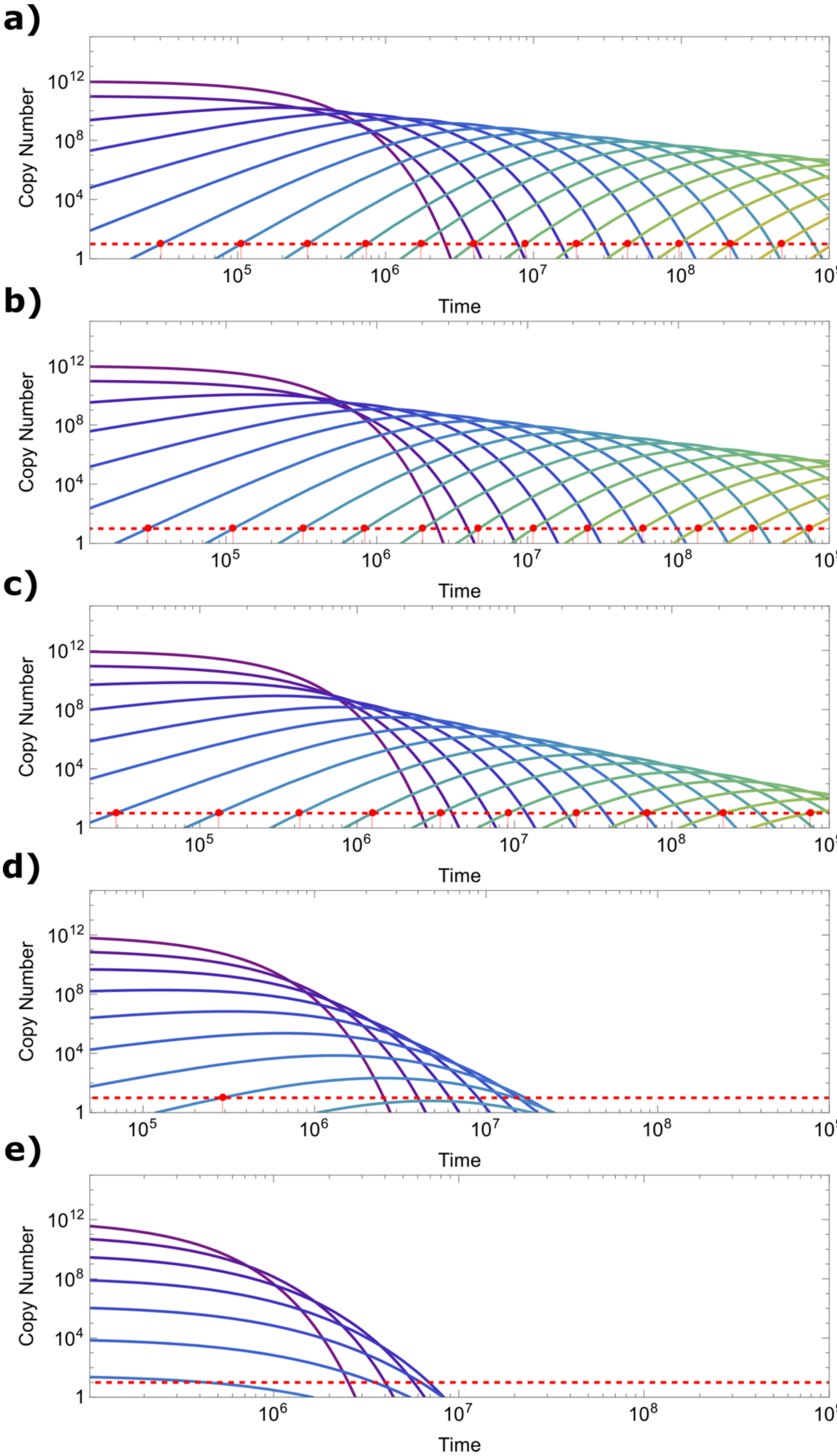


**Fig. S17 Time-dependent copy numbers with threshold time.** (a) – (e) Copy number of individual objects (assumed as equal) at different assembly indices in the range 0–25 (dark purple to orange) with for $\alpha = \{0.001, 0.2, 0.5, 0.8, 1.0\}$. The horizontal red dashed line represents threshold copy number 10, and the red point are the times at which the copy number reaches the required threshold for the first time.

Additionally, the basic discovery-production model also suggests that the nature of different physical processes where discovery and production occur simultaneously can be described by the two

characteristic time scales $\tau_d$ and $\tau_p$. Based on the observed unique objects, their assembly index and copy numbers, the Assembly ($A$) of the ensemble can be estimated from the assembly equation (1).

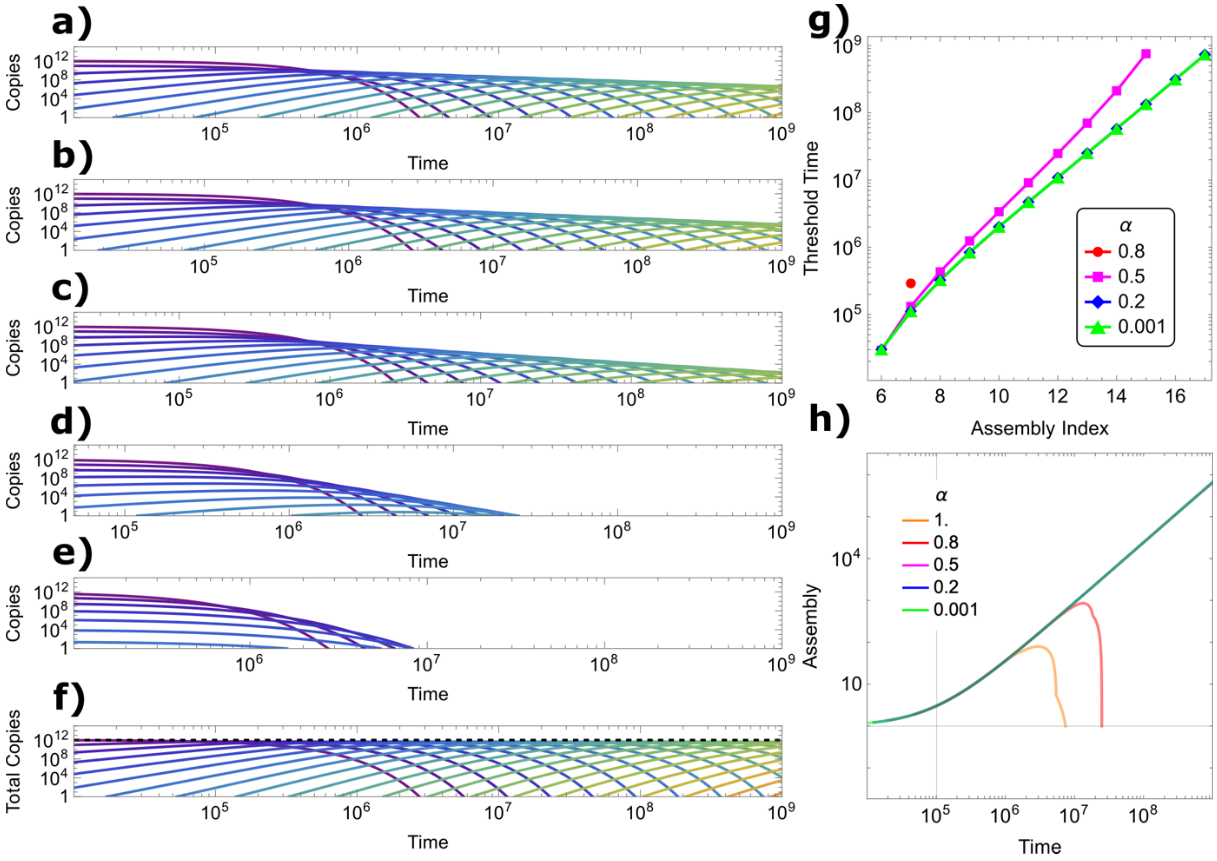


**Fig S18. Dynamics in assembly contingent coupled with kinetics.** (a)–(e) Observed copy numbers of unique objects (assumed as equal) at assembly index $a = 0 - 25$ (dark purple to orange) with $\alpha = 0.001, 0.2, 0.5, 0.8, 1.0$. (f) The total number of objects at different assembly indices, $N_a(t)n_a(t)$, $\alpha = 0.001$. (g) the threshold time to reach a copy number of 10 for various $\alpha$ values at different assembly indices starting from $a = 1$. (h) Assembly of the ensembles vs. time at various $\alpha$ values calculated using equation 1. The summation was performed over all objects from $a = 0 - 25$, where only cases when number of copies of individual objects > 1 were considered, such that the numerator of the linear term in equation 1 at assembly index $a$ is given by: $N_a(t)(n_a(t) - 1)$ if $n_a(t) > 1$. Note that $\alpha = 0.001, 0.2, 0.5$ overlap with each other which is an outcome of the simplicity of the model.

Fig. S18a–e shows the distribution of copy numbers of unique objects ($n_a(t)$) up to the dimensionless time $10^9$ for assembly indices up to 25 and for selection parameter $\alpha = 0.001, 0.2, 0.5, 0.8, 1.0$. With the increase in $\alpha$ (corresponding to a *decrease* in selectivity), the number of unique objects increases rapidly, leading to a rapid decline in the respective copy numbers of objects over time. The total copy number at all assembly indices is shown in Fig. 18f.

Fig. S18g shows the discovery time defined as the *threshold time* when the copy number of an object reaches a minimum threshold for detection (in this case we set that value to 10 copies to illustrate how this works in the discovery-production model). The threshold time depends on the limitations of

measurement (the threshold for detection of the object), the discovery and production time scales and the selection parameter $\alpha$. As an illustrative example, consider a system with fixed selectivity: a fast discovery rate means new objects can be discovered more rapidly, but a slower production rate would have the effect that it takes longer to achieve the minimal threshold copy number for detection. By contrast, with a slower discovery rate, it would take longer to discover an object, however, that could be compensated by a faster production rate where it takes much less time to achieve the threshold copy number once an object is discovered for the first time.

To show how Assembly captures when selection has driven the generation of ensembles of high complexity, we calculated $A$ for ensembles with varying selectivity $\alpha$ as shown in Fig. S18h. For a physical process with low selection capacity (high $\alpha$ values 0.8, 1.0), the production process is not sustainable and copy numbers decrease rapidly leading to a fall in Assembly over time as seen in the simplified model. With an increase in selectivity, the number of unique products is restricted, and the production process is sustainable over higher assembly values as high copy numbers are produced. In all the three cases ($\alpha = 0.001, 0.2, 0.5$), in the simplified model at all assembly indices the copy number of all objects is > 1, hence Assembly grows similarly for each case.

In this formulation, using a simplified continuum model, we combined discovery dynamics together with mass-transfer kinetics as a viable way to describe the potential nature or outcomes of a physical process with features emerging over large time scales. Here, for simplicity, we assume that the discovery dynamics (equation (8)) directs the mass transfer kinetics (equation (20)) and the rate of discovery events defined by the formation of unique products at a given assembly index is independent of the copy numbers of existing objects. In principle, to *discover* unique objects at a given assembly index $a + 1$, the discovery process must require a minimum copy number (threshold value) for objects as assembly index $a$. This effect of copy number can be introduced into the discovery equation by defining the modified discovery coefficient $\kappa_{d,m} = f(\kappa_d, n_C, n_a(t))$, where $\kappa_d$ is the concentration-independent fixed rate constant, $n_c$ is the threshold copy number, and $n_a(t)$ is the current copy number. One form of the modified discovery coefficient could have sigmoidal dependence $k_{d,m} \sim \frac{k_d}{1+e^{n_c - n_a(t)}}$, where $k_{d,m}$ increases gradually to $k_d$ depending on the copy number of the observed object at assembly $a$.

Based on the discovery coefficient ($k_d$) and mass transfer kinetics coefficient ($k_p$), the two characteristic time scales for physical processes can be defined as the discovery time scale $\left(\tau_{\mathrm{d}} \sim \frac{1}{k_d}\right)$

and production time scale $\left(\tau_p \sim \frac{1}{k_p}\right)$. So, the discovery time scale ($\tau_\mathrm{p}$) defines the characteristic time for discovering novel unique objects or as the characteristic time for the assembly process to discover novel unique objects from the assembly pool along assembly paths. In principle, the discovery timescale $\tau_\mathrm{d}$ is not necessarily constant with the assembly index and the time required to discover unique objects should also increase with the assembly index. This is primarily due to an increase in the complexity of the object, the combinatorial search space increases and more constraints emerge. The discovery process could be directed or undirected which is governed by the selection parameter ($\alpha$) and quantifies the "information processing" capacity of a physical process in selecting objects from the assembly pool. For a physical system, this is an outcome of interactions between the various objects in the assembly pool or with the external environment. The production time scale ($\tau_p$) defines the characteristic time scale for generating copies of the already discovered object along assembly paths or as the characteristic time for an *optimized process* to mass-produce the discovered object. In the case of production timescale, even for the already discovered objects at the same assembly index, $\tau_p$ could be very different for different objects leading to the observation of a high copy number of one object over the other. This concludes that for a physical system, the temporal evolution with the capacity of selection or no selection is governed primarily by three key parameters: discovery time scale ($\tau_d$), production time scale ($\tau_p$), and selection parameter ($\alpha$).

As an example, considering a physical system, with a fixed defined selection parameter ($\alpha$), the temporal evolution over the long timescale could have different outcomes depending on the discovery and production time scales. Considering three different cases,

1. $\tau_\mathrm{d} \ll \tau_\mathrm{p}$ : When the discovery time scale is far smaller than the production time scale, in that case, the discovery of unique objects is much faster leading to the fast expansion of assembly space (assuming $\alpha$ is not too small). The production process will lead to a low observed copy number of a large set of unique objects. This could lead to the formation very large set of unique objects with extremely low copy numbers eventually leading to no selection.

2. $\tau_\mathrm{d} \gg \tau_p$ : When the discovery time scale is larger than the production time scale, in that case, the assembly space expansion is slow and faster production of a smaller set of discovered objects could lead to high copy numbers. However, due to the absence of enough unique objects, the system will lack the capacity for information processing, also leading to no selection.

3. $\tau_{\mathrm{d}} \approx \tau_{\mathrm{p}}$ : In the two regimes described above, the emergent dynamics with selection and evolutionary processes are very unlikely as in the first case, assembly paths can break down due to the absence of a sufficient copy number and in the second case, outcomes can be very specific as the system lacks novelty due to lack of enough unique objects along casual paths. However, when both time scales are comparable or within a certain range, the physical system has enough flexibility to create sufficient unique objects with high enough copy numbers to move along assembly paths leading to the emergence of evolutionary processes.

## 9. Code Availability

All the calculations unless specifically mentioned were performed using Wolfram Mathematica 13. And the Mathematica Notebooks used for generating figures are available on GitHub. https://github.com/croningp/assemblyphysics.

## 10. References

1. Marshall, S. M., Moore, D. G., Murray, A. R. G., Walker, S. I. & Cronin, L. Formalising the Pathways to Life Using Assembly Spaces. *Entropy* **24**, 884 (2022).
